# Free Energy Differences from Molecular Simulations: Exact Confidence Intervals from Transition Counts

**DOI:** 10.1021/acs.jctc.2c01237

**Published:** 2023-03-16

**Authors:** Pavel Kříž, Jan Beránek, Vojtěch Spiwok

**Affiliations:** † Faculty of Mathematics and Physics, Charles University, 186 75 Prague, Czech Republic; ‡ Department of Biochemistry and Microbiology, University of Chemistry and Technology, 166 28 Prague, Czech Republic

## Abstract

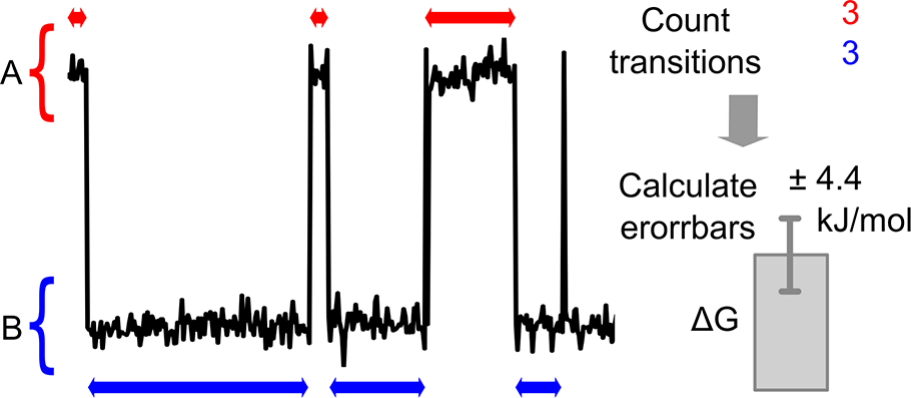

Here, we demonstrate a method to estimate the uncertainty (confidence intervals and standard errors) of free energy differences calculated by molecular simulations. The widths of confidence intervals and standard errors can be calculated solely from temperature and the number of transitions between states. Uncertainty (95% confidence interval) lower than ±1 kcal/mol can be achieved by a simulation with four forward and four reverse transitions. For a two-state Markovian system, the confidence interval is exact, regardless the number of transitions.

## 1 Introduction

One of the main purposes of molecular simulations is to predict equilibrium constants and associated free energy differences for processes, such as conformational changes, formation of noncovalent complexes, chemical reactions, or phase transitions. The equilibrium constant of a transition from state A to B can be determined experimentally as a fraction of the concentrations of B and A in equilibrium. In molecular simulations, it is common to simulate only one copy of the system (e.g., a single solvated protein or a protein–ligand pair); thus, the equilibrium constant can be predicted as a fraction of time spent in states B and A. For practical application of such predictions, it is necessary to assess their accuracy.

Accuracy of molecular simulations is determined by systematic and random errors. Systematic errors can be caused, for example, by over- or underestimation of some noncovalent interactions or oversimplification of the structure-energy relationship. In this work, we deal with random errors (uncertainties) caused by lack of sampling. They can be, in principle, eliminated by running infinitely long simulations.

Current statistical methods used in this field are based on the treatment of autocorrelation.^1−3^ Many characteristics of a molecular system, including the densities of states, can be calculated by averaging along the trajectory. Accuracy of the mean value can be characterized by a standard error of the mean. It is commonly calculated as , where σ is the standard deviation and *n* is the number of samples. However, *n* can be set to the number of samples only if these samples are independent. This is not the case of states sampled along a simulation trajectory because they are strongly autocorrelated. To apply to a simulation trajectory, it is necessary to estimate the number of independent samples *n*, which is significantly lower than the number of all samples, either by block averaging^1,2^ or autocorrelation analysis.^3^

As an alternative, we present a simple method “JumpCount” based solely on the number of observed transitions between states of the system. The prerequisite of the method is that the states of the system can be clearly distinguished, there is an energy barrier between them, and the process is Markovian, i.e., the probability of transition from one state to another is constant and independent of the history of the system, and it can be studied by the first order kinetics. Finally, the simulation is sampled with a frequency allowing to capture all transitions.

The method is demonstrated on model data (random numbers with an appropriate statistical distribution) and two types of molecular systems–glycerol and fast-folding miniproteins.

## 2 Theory

**Figure 1:**
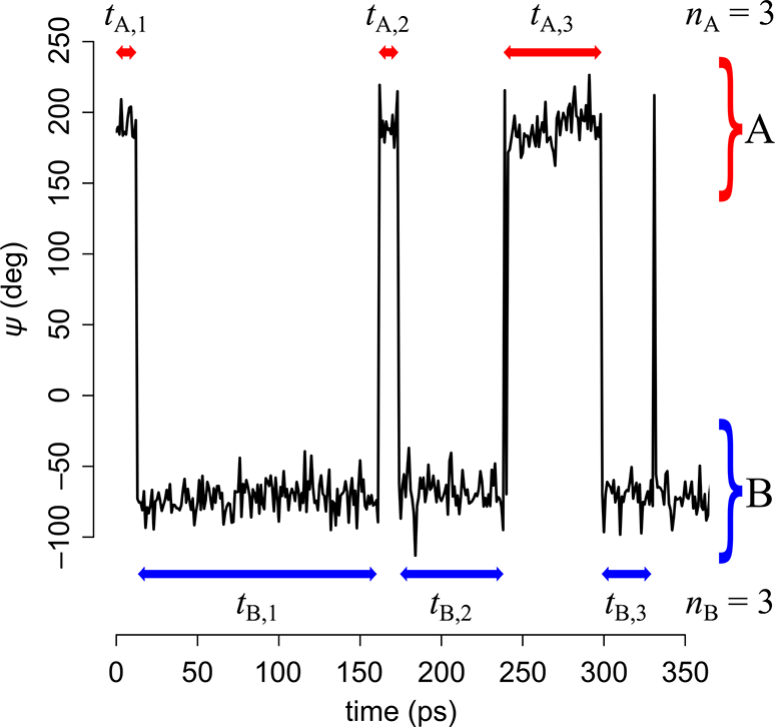
A schematic representation of a trajectory with three A to B and three B to A transitions (data taken from glycerol simulation).

First, we will derive an expression of the uncertainties of an equilibrium constant and the associated free energy difference for a simple reversible transition from state A to state B. A simulation can be performed in a way that undergoes the same number of transitions from A to B (*n*_A_) and from B to A (*n*_B_). This is illustrated in Figure 1 for the number of transitions *n*_A_ = *n*_B_ = 3.

For a Markovian process, the first time passage times for the transition from A to B (and vice versa) are exponentially distributed with the probability *P*(*t*_A_) = *k*_1_ exp(−*k*_1_*t*_A_), where *k*_1_ is a rate constant for the transition from A to B. Analogously, *P*(*t*_B_) = *k*_–1_ exp(−*k*_–1_*t*_B_). It is possible to estimate *k*_1_ and *k*_–1_ as *n*_A_/*∑*_*i*_*t*_A,*i*_ and *n*_B_/*∑*_*i*_*t*_B,*i*_, respectively.

The sum of independent random variables with exponential distribution follows the gamma distribution, namely, Gamma (shape = *n*_A_, rate = *k*_1_) for . The equilibrium constant *K̂* can be estimated as  (*K̂* will be used as a symbol for an estimate of the true equilibrium constant *K*). The corresponding free energy difference can be estimated as , where *k* is Boltzmann constant and *T* is the temperature in Kelvins. The fraction of the estimate and the true value of *K* can be written as a rescaled fraction of independent gamma-distributed random variables and follows the F-distribution (also known as Fisher-Snedecor distribution, known from F-test and ANOVA)^4^ with degrees of freedom *d*_1_ = 2*n*_B_ and *d*_2_ = 2*n*_A_. The confidence interval (CI) of *K* can therefore be calculated using the quantile function of the F-distribution. The 95% CI of *K* can be calculated as
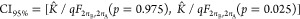
1where *qF* is the quantile of *F*-distribution (inverse of the cumulative distribution function) with subscripts *d*_1_ and *d*_2_.

For free energy, the 95% CI can be calculated as
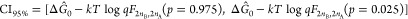
2

As a result, the CI of the free energy difference depends solely on temperature and the number of transitions. For 300 K, the 95% CI for the free energy difference is  (2.18 kcal/mol) for *n*_A_ = *n*_B_ = 1,  (1.35 kcal/mol) for *n*_A_ = *n*_B_ = 2, etc. (see Table 1). The confidence interval , which is often used as a threshold of accuracy in molecular simulations, can be reached in a simulation with *n*_A_ = *n*_B_ = 4.

**Table 1:**
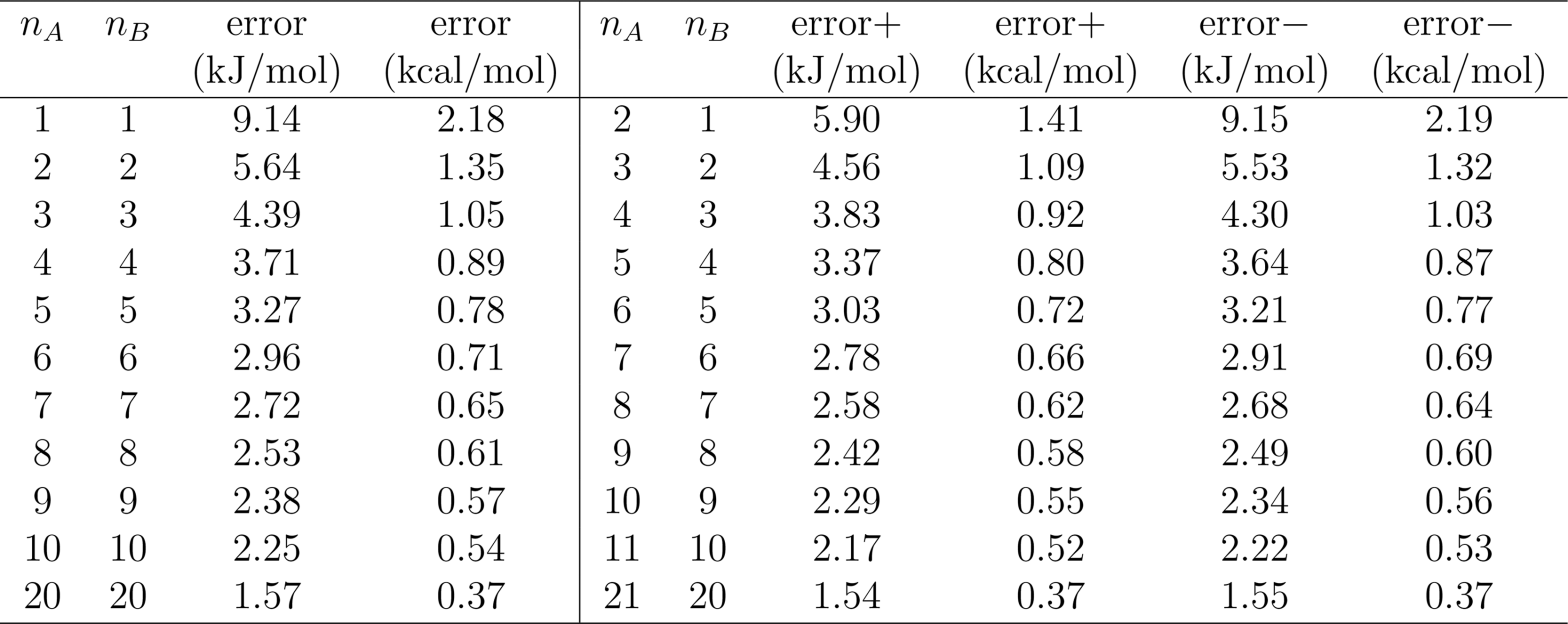
95% Confidence Intervals at 300 K (Mean ± Error) for Processes with Numbers of Transitions from A to B (*n*_A_) and from B to A (*n*_B_)

The concept described above can be easily generalized to *n*_A_ = *n*_B_ + 1 (we denote the starting state as A). In this case, the confidence interval can be calculated by setting the degrees of freedom *d*_1_ = 2*n*_B_ and *d*_2_ = 2*n*_A_ in the *F*-distribution. The confidence interval is then asymmetric (see Table 1).

The variance of Δ*G*_0_ can be calculated as
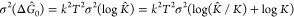
3Since *K* is exact (its variance is 0) and *K̂*/*K* follows *F*-distribution, the variance of  and standard error can be calculated as
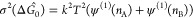
4
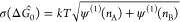
5where ψ^(1)^ is a polygamma function of order 1 (trigamma). The values of standard error are presented in Table 2. However, we believe that confidence intervals are more informative because the free energy estimates discussed here are not normally distributed and most researchers are familiar with standard errors in the context of normally distributed samples.

**Table 2:**
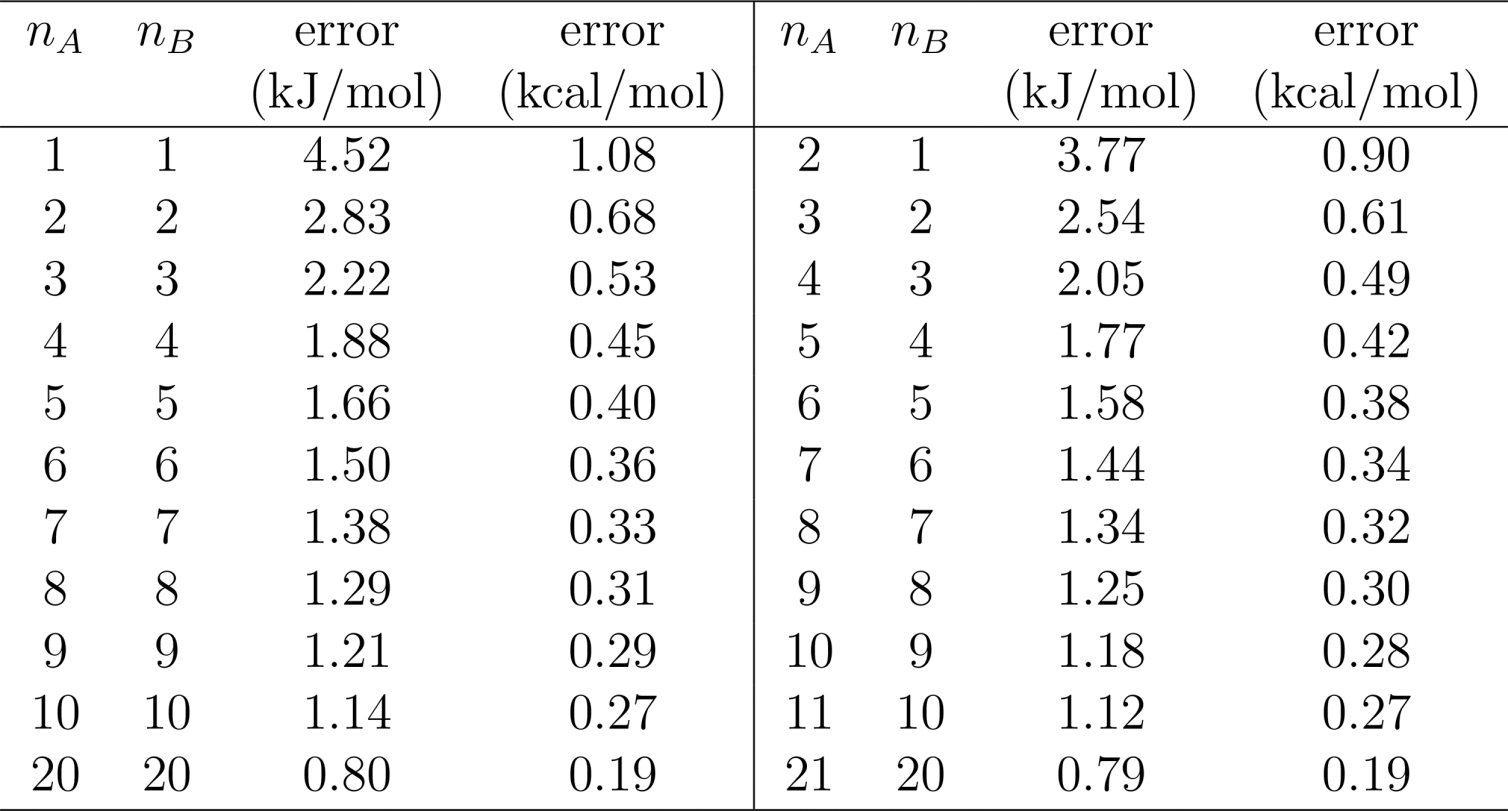
Standard Errors at 300 K (Mean ± Error) for Processes with Numbers of Transitions from A to B (*n*_A_) and from B to A (*n*_B_)

For a system with multiple states (e.g., A, B, and C), it is possible to calculate confidence intervals and standard errors as described above, however, it is necessary to count only the accomplished transitions between states. For example, when calculating Δ*G*_A→C_ it is necessary to count the process with transitions A → B → A → B → C as a single accomplished transition from A to C. The resulting numbers of accomplished transitions *n*_A_ and *n*_C_ can be used in eqs 1 and 2 to obtain CI of *K*_A→C_ and Δ*G*_A→C_ (see Supporting Information for a demonstration of the fact that the sum of *t*_A*i*_ and the sum of *t*_C*i*_ follow the Gamma(shape = *n*_A_, ...) and Gamma(shape = *n*_C_, ...) distributions, respectively).

As an alternative, for a system with multiple states, it is possible to calculate the free energy differences for states with direct transitions (e.g., A to B and B to C, for a system with A ⇌ B ⇌ C transitions) separately and combine errors as . This approach is rigorous for variables with normal distribution. It is possible to apply it to systems with a high number of transitions, because the distribution of *K̂*/*K* becomes a close-to-normal distribution according to the central limit theorem.

## 3 Computational Details

Numerical simulations with random numbers were performed in R 3.4.4.^5^ Molecular dynamics simulation of gylcerol in water was conducted using GROMACS 2018.6. Relevant preparation steps were conducted using GROMACS 2022.3.^6^ The preparation of the system for MD simulation was as follows: Starting structure of *sn*-glycerol was obtained by conversion of SMILES file to PDB format using Open Babel.^7^ Topology was built manually according to Glycam06.^8^ Partial atomic charges were calculated at HF/6-31G*//HF/6-31G* level of the theory using the RESP method.^9^ Simulation box was cubic with a size of 2.94013 nm. The molecule was solvated by TIP3P water. No ions were added, as the net charge of the system was zero. Potential energy of the system was then minimized using the steepest descent method, until the maximum force acting on any atom was lower than 1000 kJ/mol/nm. This step was followed by isothermal-isochoric equilibration and isothermal–isobaric equilibration, each at 300 K for 100 ps.

Then, 1 μs long MD simulation was conducted. At the beginning of the simulation, the velocities of atoms were generated randomly from Maxwell distribution for 300 K. In the relevant steps of equilibration and MD run, the following parameters were used: Leapfrog integrator (md), radius for short-range electrostatic and van der Waals was set to 1 nm (spanning the whole molecule of glycerol). Particle Mesh Ewald method^10^ was used for computing long-range electrostatic interactions. Temperature coupling was conducted using Parrinello–Bussi thermostat^11^ (300 K) and pressure coupling was conducted with Parrinello–Rahman barostat^12^ (1 bar). After obtaining the trajectory of glycerol from the MD run, the values of torsion angles in the molecule were computed at each 1 ps using Plumed.^13^

Trajectories of fast-folding miniproteins were taken from literature.^14^

## 4 Results

**Figure 2:**
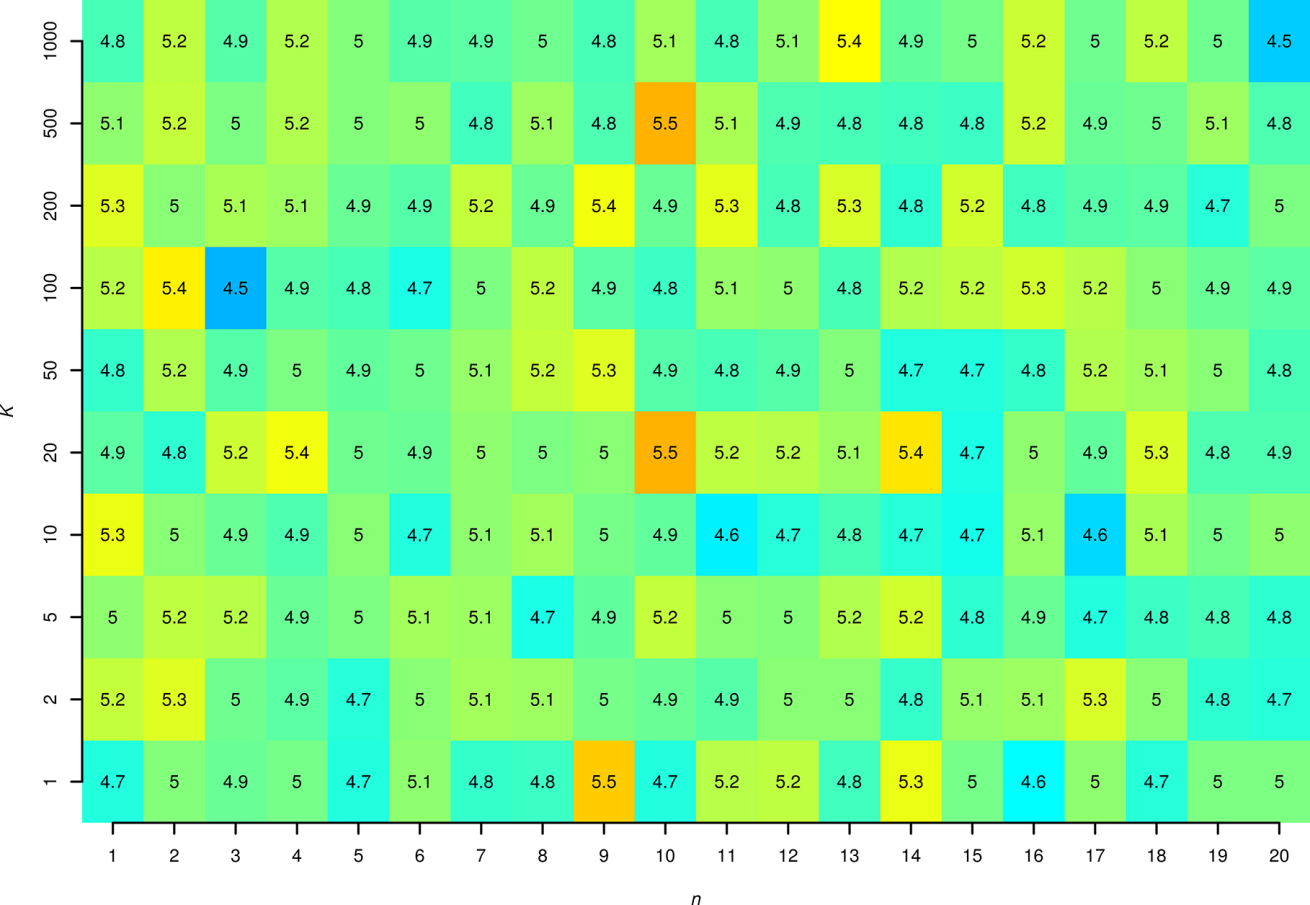
Rates of type 1 errors (in %) for different *n*_A_ = *n*_B_ and *K* in simulations using generated random numbers with exponential distribution.

The easiest way to test the concept is to generate the first time passage times as exponentially distributed random numbers. This was performed in the R software (see Supporting Information). We tested scenarios with *n*_A_ = *n*_B_ set to 1 to 20 and with *K* set to 1 to 1,000. The values of *k*_–1_ and *k*_1_ were set to 1 and *K*, respectively (in arbitrary units). For each pair of *n* and *K* we generated 10,000 sets of first time passage times and calculated 95% CI of *K* and 95% CI of the free energy difference (by eqs 1 and 2, respectively). We expect the rate of type I errors (i.e., the fraction of trials for which the predefined *K* lies outside the calculated CI) to be 5%. Indeed, the type I error rate ranged from 4.50 to 5.52% with a median of 4.99% (Figure 2). Similar results were observed for *n*_A_ = *n*_B_ + 1 (see Supporting Information).

**Figure 3:**
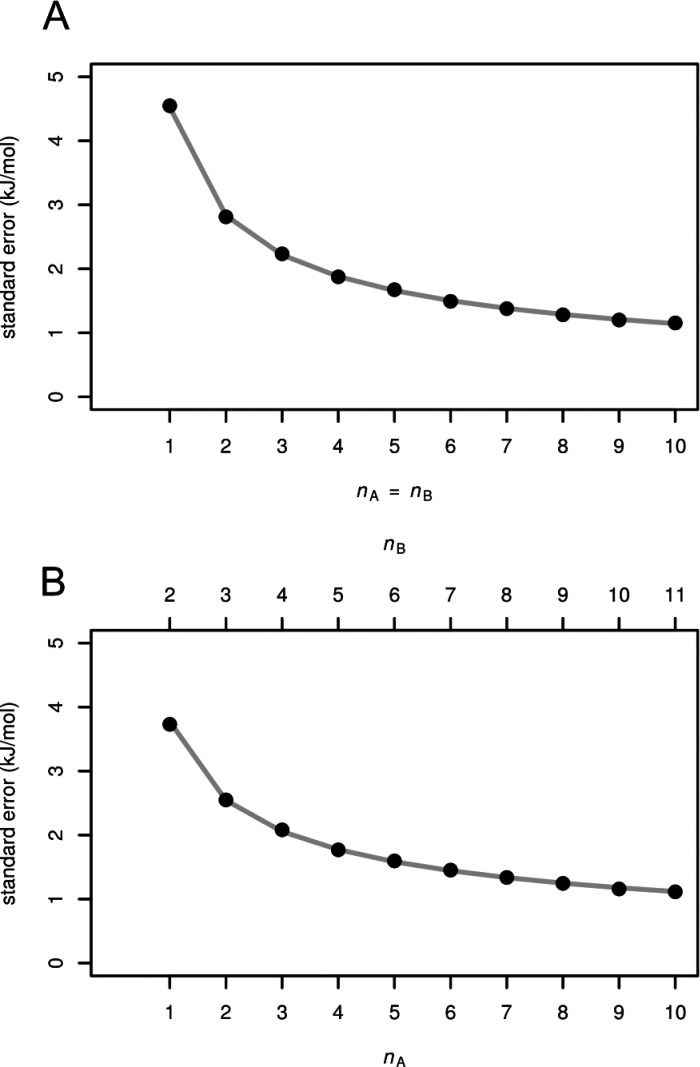
Standard errors calculated by eq 5 (lines) and numerically (points) for *n*_A_ = *n*_B_ (A) and *n*_B_ = *n*_AB_ + 1 (B).

Numerical support for eq 5 for calculation of standard errors is demonstrated in Figure 3. For each *n*_A_ and *n*_B_ we generated *n* × 10,000 sets of first time passage times and calculated standard error by eq 5 and numerically as a standard deviation of 10,000 calculated values of Δ*G*_0_. There is a perfect agreement.

Our approach was demonstrated on two types of molecular systems. An example of a molecular system with multiple states is a glycerol molecule in water.^15^ Each of the two O–C–C–O torsion angles can adopt three minima. This gives nine combinations; however, the three pairs are equivalent as a result of the symmetry of the molecule; thus, six conformers can be experimentally resolved. Equilibria of these conformers have been studied by molecular simulations as well as experimentally.^15,16^

Here, we performed a 1 μs simulation of glycerol in water and calculated the equilibria for six conformers. Consistent with simulation and experimental studies^15,16^ we observed conformer populations *αγ* > *αβ* > *αα* > *βγ* > *γγ* > *ββ* ∼ *γγ*. Confidence intervals for all conformers relative to *αγ* were calculated at times 5, 10, 20, 50, 100, 200, 500, and 1,000 ns. In total, 37 confidence intervals were calculated (eight for each conformer except *αγ*, confidence intervals for *γγ* were not available at 5, 10, and 20 ns due to no sampling). These confidence intervals were compared with the value of Δ*G* calculated from the whole trajectory. Since we do not know the exact value of Δ*G*, we used this value as a “ground truth”.

**Figure 4:**
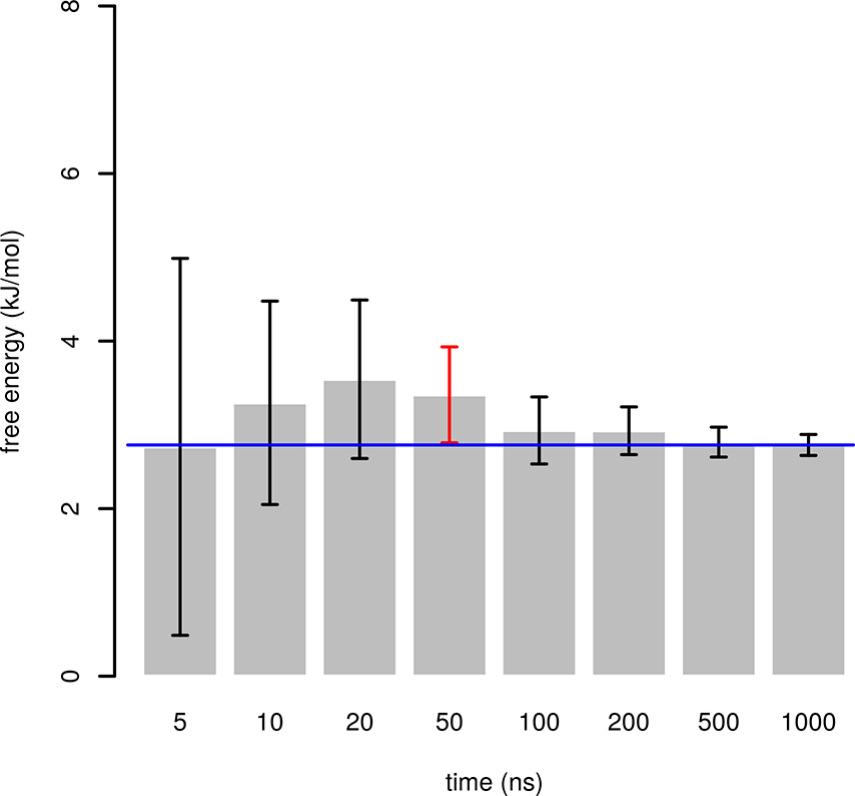
Confidence intervals of free energy of *βγ* conformer of glycerol, relative to *αγ*. The value of free energy calculated for the whole simulation is depicted as a blue line. A confidence interval that does not span this value is depicted in red.

One of these 37 confidence intervals was not spanning the ground truth Δ*G*. This was the case of Δ*G* of *βγ* at 50 ns (Figure 4). The distance of Δ*G* from the confidence interval was very low. Figures for other conformers are available in Supporting Information. Since we compare the confidence intervals with the estimate of Δ*G* calculated for whole trajectory, not the exact value of Δ*G*, we expect the rate of type 1 errors to be lower than 5%. This is in agreement with the observed 1/37 (2.7%).

The main prerequisite of the above-outlined approach is Markovianity of the processes studied. This is usually fulfilled for transitions associated with a single energy barrier, such as simple conformational changes or chemical reactions. More complex transitions, such as protein folding, can be non-Markovian.^17^ The method of molecular dynamics simulation is Markovian because each state of the molecular system depends solely on the previous state, not on the history. However, coarse graining of the system representation into a few substates (e.g., folded and unfolded, ligand-bound, and unbound) and ignoring the complex kinetics within these states may cause the Markovianity condition to be not fulfilled.

Keeping in mind the limitation of the Markovianity prerequisite, we applied our approach to the folding and unfolding trajectories of fast folding mini-proteins.^14^ Trajectories were kindly provided by D.E. Shaw research. Root-mean-square deviation (RMSD) profiles from the native structure were calculated, and folded and unfolded states were assigned by visual inspection of RMSD profiles and trajectories. Folding free energies were estimated as  for the whole trajectories (multiple trajectories of the same system were combined). These values were used as a ground truth.

**Figure 5:**
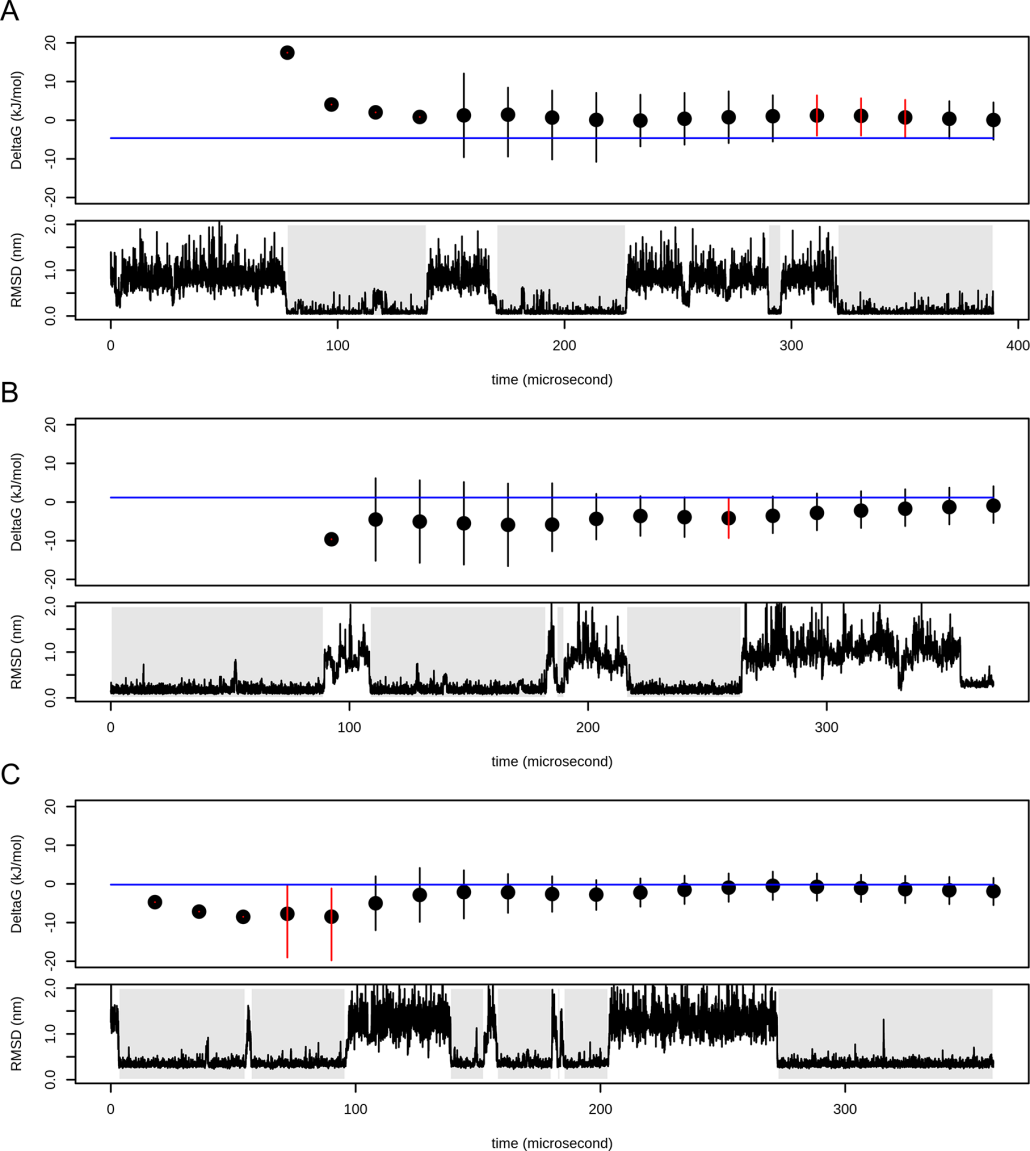
Confidence intervals of folding free energies for (A) NTL9 (simulation 2), (B) protein G (simulation 0), and (C) α3D (simulation 1). Top part of each subplot shows calculated folding free energy with confidence intervals depicted as error bars. The value of folding free energy calculated for the whole simulation is depicted as a blue line. Confidence intervals that do not span this value are depicted in red. Bottom parts show RMSD profiles. Folded states are highlighted by gray background.

They were compared with  calculated at 20 points equally distributed along each trajectory. The ground truth was outside the 95% confidence intervals for six of 432 (1.39%) values. These confidence intervals are shown in Figure 5. Corresponding plots for other systems can be obtained in Supporting Information.

Since we do not know the exact value of Δ*G*_folding_ and we used the value calculated for the whole simulation as the ground truth, we expect a lower number of type 1 errors (values outside 95% CI) than 5%, which is in good agreement with the observed 1.39%. Furthermore, all values outside the confidence intervals were located very close to them.

## 5 Discussion

Our results show that relatively accurate predictions of Δ*G* can be obtained from simulations with relatively low number of transitions between the states. This finding may reduce the costs and increase the efficiency of future applications of molecular simulations in ligand design and other fields.

Most important, in our opinion, is the fact that the method is very easy to use. It requires counting of transitions between states of the system and looking up confidence intervals from a table or by a simple program. It must be kept in mind that automated identification of transitions between states can be challenging. While for the glycerol system presented above it was possible to decide the state based on torsion angles, for fast-folding miniproteins, it was necessary to count transitions “manually” based on visual inspection of the trajectories, because the value of RMSD from the native structure cannot strictly distinguish the folded and unfolded states.

It is possible to generalize the concept to binding, such as simulation of protein–ligand interactions. The dissociation constant of a complex PL (the equilibrium constant of PL ⇌ P + L) can be expressed as *K*_d_ = *c*_L_*∑t*_P_/*∑t*_PL_, where *c*_L_ is the concentration of the ligand in the simulation box in the unbound state. The value of Δ*G*_0_ is calculated as *kT*  log  *K*_d_. Therefore, the accuracy of *∑t*_P_/*∑t*_PL_ (and thus for the resulting *K*_d_ and binding Δ*G*_bind_) can be calculated as described above.

The concept described above was presented for one long simulation with multiple transitions between the states of the system. Another design of molecular simulations can be to run a series of *n*_A_ independent simulations starting from the state A until they transit to state B and a series of *n*_B_ simulations starting from state B until they transit to the state A. The value of *K* can be estimated as
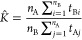
6The confidence intervals and standard errors can be estimated by the equations presented above.

However, we must warn readers who are not patient enough to run all simulations until all transitions are observed. The method described above can be used only when all simulations result in transitions from A to B or from B to A.

Let us consider the situation when 10 simulations starting from A are performed, but only one of them (*i* = 1) results in a transition to B. Other 9 simulations end at time *t*_max_ without a transition. Some users might be tempted to estimate the rate constant *k*_1_ as 1/*t*_*A*1_ and to discard the results of 9 unsuccessful simulations. However, *k*_1_ is likely to be significantly overestimated because 9 of the 10 simulations did not reach state B. The correct estimator of *k*_1_ with unfinished simulations (right-censored *t*) is
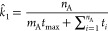
7where *n*_A_ is the number of simulations in which the transition from A to B was observed (indexes *i*) and *m*_A_ is the number of simulations in which the time *t*_max_ is reached without a transition.

Therefore, *K* can be estimated as
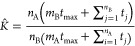
8Derivation of equations for this design of simulations is presented in Supporting Information. The fact that some long simulations starting from state A fail to reach state B, while others reach it quickly, can also be a signature of non-Markovianity.

The results show that our approach can be applied to fast-folding mini-proteins. Either the degree of non-Markovianity in these systems is not high enough to significantly affect the performance of our approach or this approach is robust enough for the non-Markovianity typical for biomolecular systems.

In principle, non-Markovianity can be tested by inferential statistics methods, such as by Kolmogorov–Smirnov test (or some other goodness-of-fit test) to assess the deviation from the exponential distributions. However, we would like to stress that the power of the Kolmogorov–Smirnov test is rather low, i.e., it can reject Markovianity provided that there are enough samples (enough transitions). With few transitions, the test neither rejects nor approves Markovianity.

The problem of non-Makovianity can be solved by dissection of sampled states into a minimal set of substates for which mutual transitions are Markovian. This approach is used when building Markov state models.^18^ Alternatively, it would be possible to estimate the true distribution of *t*_A_ and *t*_B_ for non-Markovian systems and derive corresponding distributions for *K* and Δ*G*.

We argue that the approach can be generalized to biased simulations with a static bias potential, such as single-replica umbrella sampling.^19^ Adapting the approach to methods with a time-dependent bias potential, such as metadynamics,^20^ will be the subject of future research.

In Supporting Information, it is possible to find commands to calculate confidence intervals and standard errors in various programming languages. Data necessary to reproduce all calculations are available online via Zenodo (dx.doi.org/10.5281/zenodo.7610654). Online calculator of CI and standard errors is available at https://jumpcount.cz.

## Supporting Information Available

Testing of the method on random numbers with exponential distribution, detailed results of simulation of aqueous glycerol, full results of application of the method on fast-folding miniproteins, and instructions to calculate errors in various programming languages (PDF)
