# Supplementary material for: Free Energy Differences from Molecular Simulations: Exact Confidence Intervals from Transition Counts
Source: J Chem Theory Comput. 2023 Mar 16;19(7):2102–8. doi: 10.1021/acs.jctc.2c01237 (PMC10100533; doi:10.1021/acs.jctc.2c01237)
Supplement: Supplementary file 1 — ct2c01237_si_001.pdf [file ct2c01237_si_001.pdf]

Supporting Information: Free Energy Differences from Molecular Simulations: Exact Confidence Intervals from Transition Counts

Pavel Kříž,[†] Jan Beránek,[‡] and Vojtěch Spiwok[*,‡]

†*Faculty of Mathematics and Physics, Charles University, Prague, Czech Republic*

‡*Department of Biochemistry and Microbiology, University of Chemistry and Technology, Prague, Czech Republic*

E-mail: spiwokv@vscht.cz

Phone: +420 220 44 3028

1 Simulation using generated random numbers

Simulations using generated random numbers were performed in R. First, we wrote a function to generate a set of t_A and t_B using function `rexp`. It generated $n_A = n_B$ number of t_A and t_B with predefined values of k_1 set to K and k_{-1} set to 1. Next, CIs were calculated and returned to the output of the function.

The function was applied 10,000 times for each combination of $n_A = n_B$ from 1 to 20 and K set to 1, 2, 5, 10, 20, 50, 100, 200, 500 and 1000. Fractions of trials for which K is located outside CI are plotted in the Figure 2 and Figure S1 as a heatmap. The results are in good agreement with the expected rate of type 1 errors (5 % for 95-% CI).

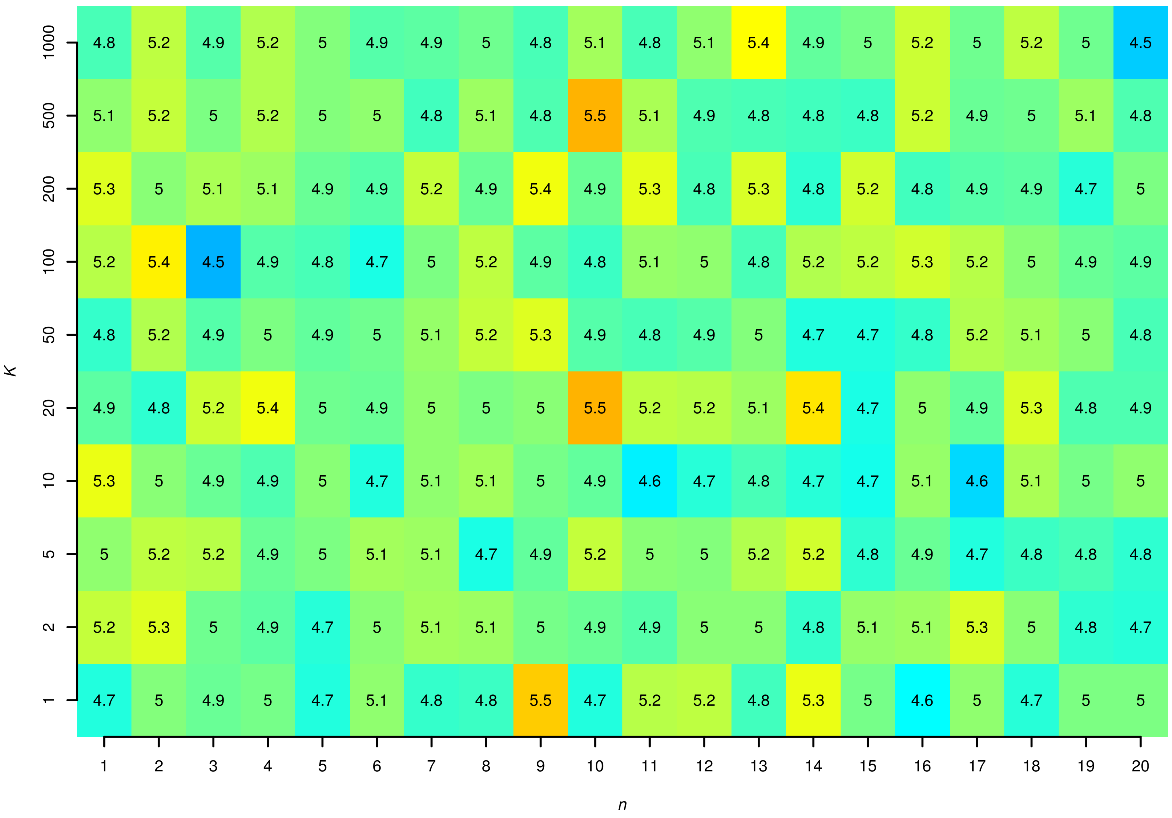

Figure S1: (identical to Figure 2 of the article) Rates of type 1 errors (in %) for different $n_A = n_B$ and K in simulations using generated random numbers with exponential distribution.

The same simulations as described above were performed with $n_A = n_B + 1$. The function was applied 10,000 times for each combination of n ($n_A = n_B + 1 = n + 1$) from 1 to 20 and K set to 1, 2, 5, 10, 20, 50, 100, 200, 500 and 1000. Fractions of trials for which K is located outside CI are plotted in the Figure S2 as a heatmap. Again, the results are in good agreement with the expected rate of type 1 errors (5 % for 95-% CI).

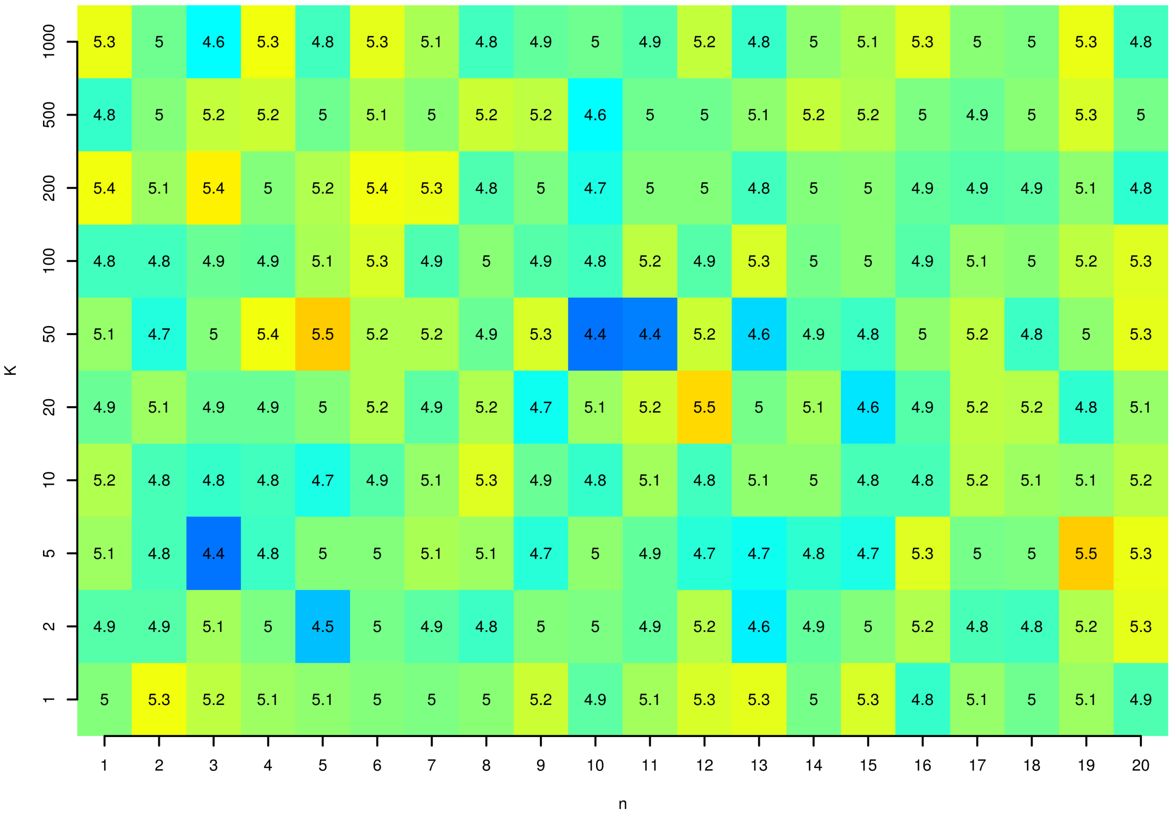

Figure S2: Rates of type 1 errors (in %) for different n ($n_A = n_B + 1 = n + 1$) and K in simulations using generated random numbers with exponential distribution.

The reasoning that Equation 1 and 2 are correct also for the three-state system with transitions A $\rightleftharpoons$ B $\rightleftharpoons$ C (with n_A and n_C being the numbers of accomplished transitions from A to C and from C to A, respectively): Take an example of a continuous-time Markov chain with possible transitions A $\rightarrow$ B with the rate constant $k_1 = K_{AB} > 0$, B $\rightarrow$ C with the rate constant $k_2 = K_{BC} > 0$, C $\rightarrow$ B with the rate constant 1 and B $\rightarrow$ A with the rate constant 1 (rate constants equal to equilibrium constants and 1 were used for simplicity; however, it can be generalized for any values).

Furthermore, consider an example of a single accomplished transition A $\rightarrow$ B $\rightarrow$ A $\rightarrow$ B $\rightarrow$ C, with the number of visits of state A being $N_A = 2$ and the total time spent in state A being $T_A = t_{A1} + t_{A2}$, where t_{Ai} is the occupation time in state A during i-th visit.

Firstly, note that the number of visits N_A of state A within a single acomplished transition is a random variable having geometric distribution Ge$[p = k_2/(1+k_2)]$. This is because every time the process visits state B, it moves either to state A (with probability $1/(1 + k_2)$) or to state C (with probability $k_2/(1 + k_2)$).

Next, individual occupation times t_{Ai} are independent random variables that have an exponential distribution with the rate constant k_1, and they are independent of N_A. Now, the total occupation time in a state A during a single accomplished transition $T_A = t_{A1} + t_{A2} + ... + t_{AN_A}$ has a compound distribution and we can show (using the machinery of moment-generating functions) that it is again an exponential distribution with intensity $k_1k_2/(1 + k_2)$. Similarly, we can demonstrate that the total occupation time in state C during a single accomplished transition from C to A has an exponential distribution with intensity $1/(1 + k_2)$.

Now the reasoning follows the same lines as in the 2-state case: the sums of total occupation times in A and C over all accomplished transitions are independent and follow the Gamma distributions Gamma$(\text{shape} = n_A, \text{rate} = k_1k_2/(1 + k_2))$ and Gamma$(\text{shape} = n_C, \text{rate} = 1/(1+k_2))$, respectively. Their ratio (denote $\hat{K}$) is the estimator of the stationary

probabilities ratio, and the expression

$$\hat{K}\,\frac{n_A}{n_C}\,\frac{1}{k_1 k_2} = \frac{\hat{K}}{K_{AB}K_{BC}} = \frac{\hat{K}}{K_{AC}}$$

has Fisher-Snedecor distribution $F(2n_C, 2n_A)$. From this, it easily follows that Equation (1) provides the exact confidence interval for the unknown quantity $k_1 k_2 = K_{AB}K_{BC} = K_{AC}$.

Simulations with three states with transitions A $\rightleftharpoons$ B $\rightleftharpoons$ C were also tested numerically. First, we wrote a function to generate a set of t. For each application of this function, the simulation starts from the state A. The value of $t_{A\to B}$ was generated and the state changed to B. Next, values of $t_{B\to A}$ and $t_{B\to C}$ were generated. If $t_{B\to C} < t_{B\to A}$, the state changed to C, otherwise it changed to A. Analogously, a pair of t was generated for any state until the desired number of accomplished transitions from A to C and C to A was observed. For example, it is necessary to count the process with transitions A $\to$ B $\to$ A $\to$ B $\to$ C as a single accomplished transition from A to C.

Confidence intervals for K were calculated as:

$$\mathrm{CI}_{95\%} = [\hat{K}/qF_{2n_C,2n_A}(p = 0.975), \hat{K}/qF_{2n_C,2n_A}(p = 0.025)]. \tag{1}$$

where n_A and n_C are the numbers of accomplished transitions from A to C and C to A, respectively.

The function was applied 10,000 times for each combination of $n_A = n_C$ set to 1, 2, 5, 10, 20 and 50 and $K_{A\to B}$ set to 10 and $K_{B\to C}$ was set to 1, 2, 5, 10 and 20.

Fractions of trials for which $K_{A\to C} = K_{A\to B}K_{B\to C}$ is located outside CI are plotted in the Figure S3 as a heatmap. Again, the results are in good agreement with the expected rate of type 1 errors (5 % for 95-% CI).

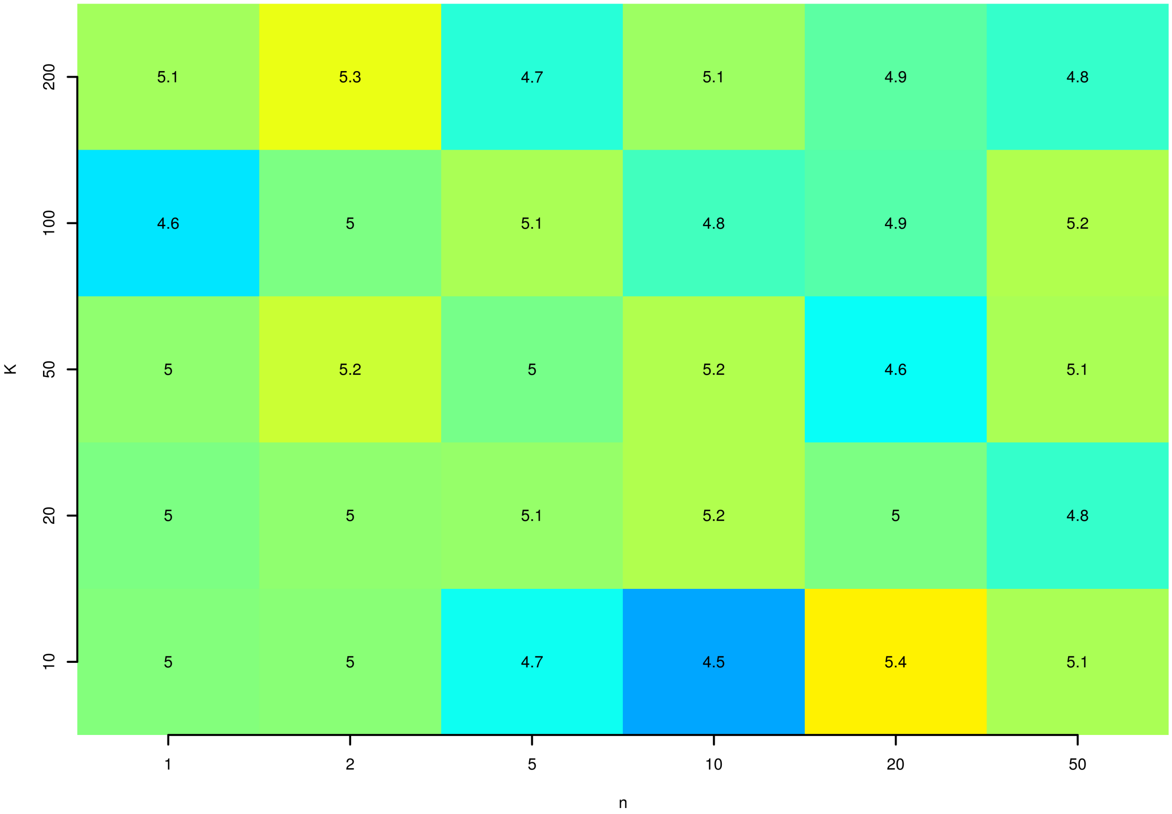

Figure S3: Rates of type 1 errors (in %) for different $n_A = n_B$ and $K_{A \to C}$ in simulations using generated random numbers with exponential distribution for a system with states A, B and C.

2 Glycerol in Water

2.1 Results

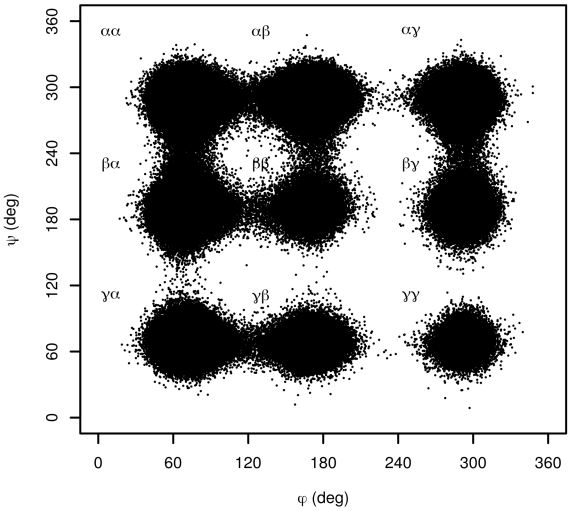

Figure S4: Torsion angles ϕ and ψ sampled during the simulations.

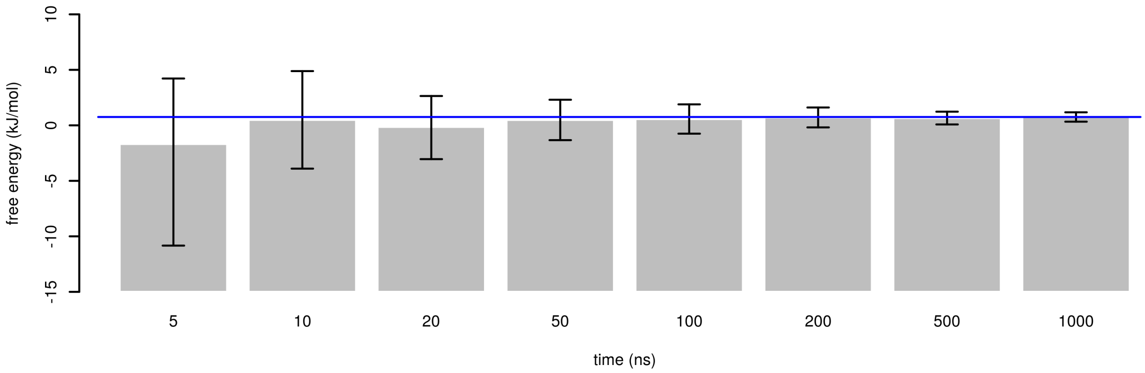

Figure S5: Confidence intervals of free energy of $\alpha\alpha$ conformer of glycerol, relative to $\alpha\gamma$. The value of free energy calculated for the whole simulation is depicted as a blue line.

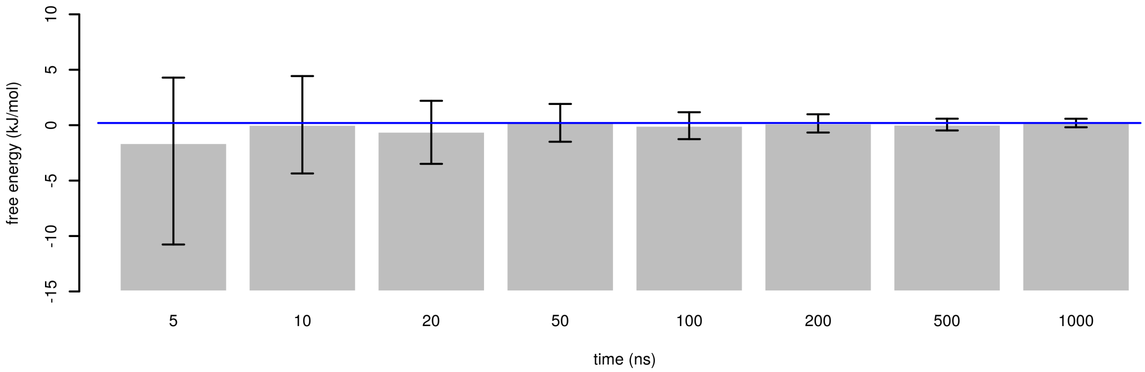

Figure S6: Confidence intervals of free energy of $\alpha\beta$ conformer of glycerol, relative to $\alpha\gamma$. The value of free energy calculated for the whole simulation is depicted as a blue line.

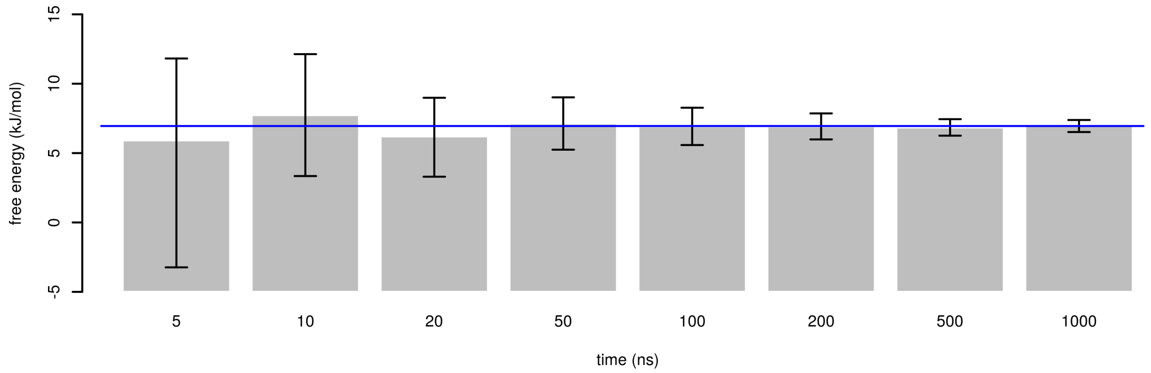

Figure S7: Confidence intervals of free energy of $\beta\beta$ conformer of glycerol, relative to $\alpha\gamma$. The value of free energy calculated for the whole simulation is depicted as a blue line.

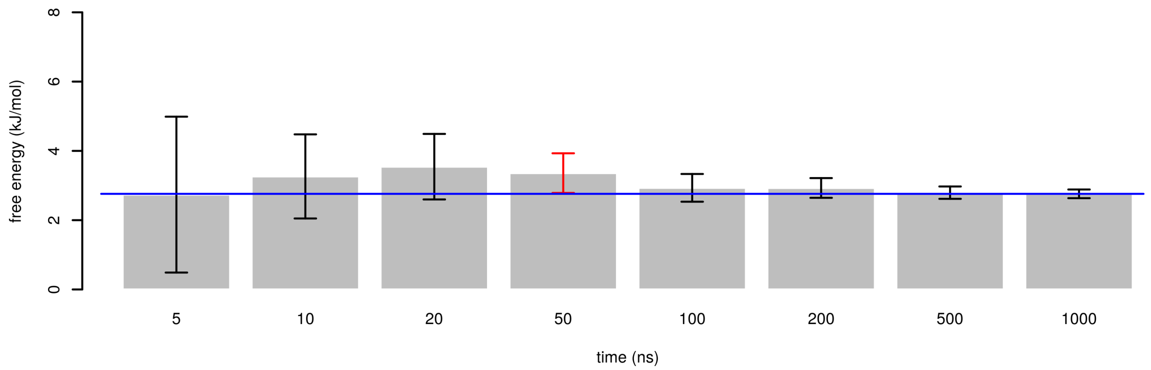

Figure S8: Confidence intervals of free energy of $\beta\gamma$ conformer of glycerol, relative to $\alpha\gamma$. The value of free energy calculated for the whole simulation is depicted as a blue line. A confidence interval that does not span this value is depicted in red.

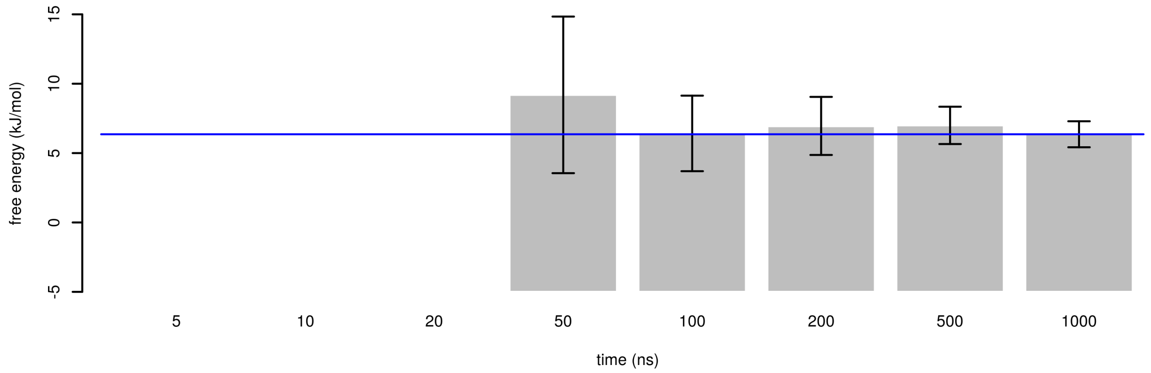

Figure S9: Confidence intervals of free energy of $\gamma\gamma$ conformer of glycerol, relative to $\alpha\gamma$. The value of free energy calculated for the whole simulation is depicted as a blue line.

3 Fast folding miniproteins

3.1 Results

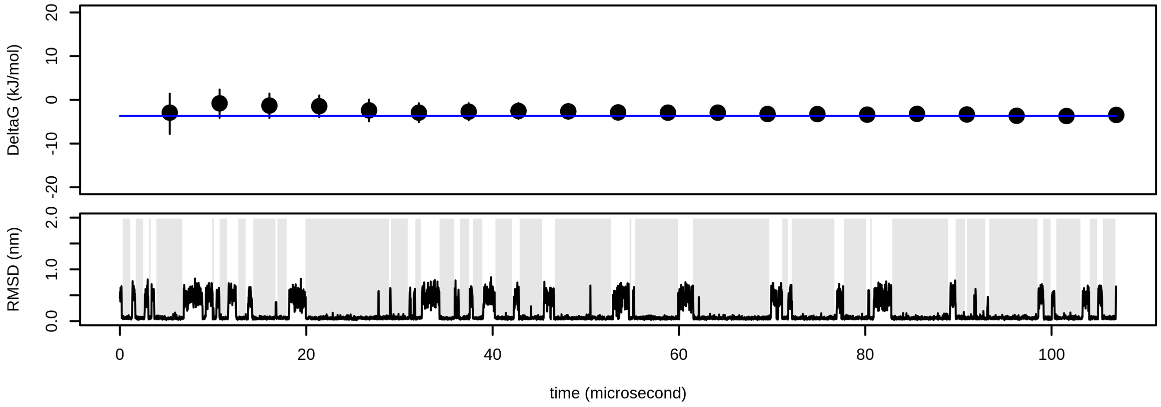

Figure S10: Confidence intervals of folding free energy of Chignolin. The value of free energy calculated for the whole simulation is depicted as a blue line.

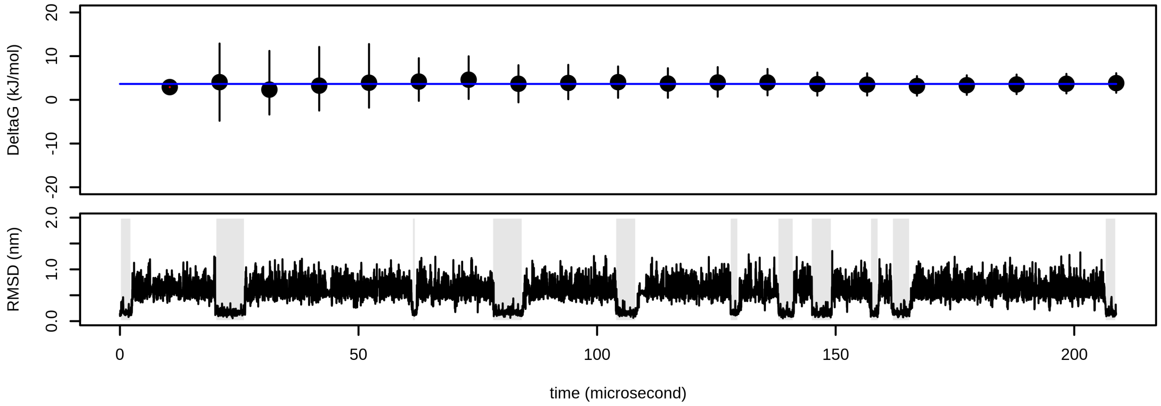

Figure S11: Confidence intervals of folding free energy of Trp-cage. The value of free energy calculated for the whole simulation is depicted as a blue line.

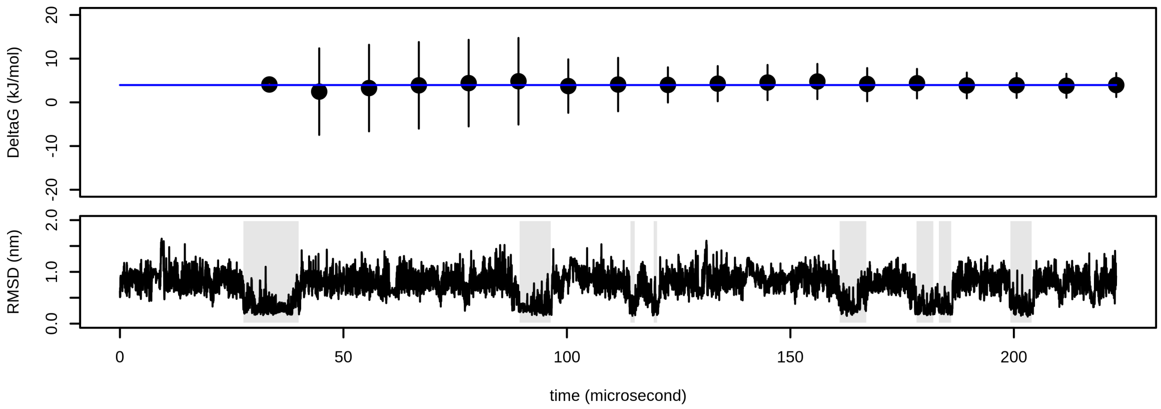

Figure S12: Confidence intervals of folding free energy of BBA (simulation 0). The value of free energy calculated from all simulations is depicted as a blue line.

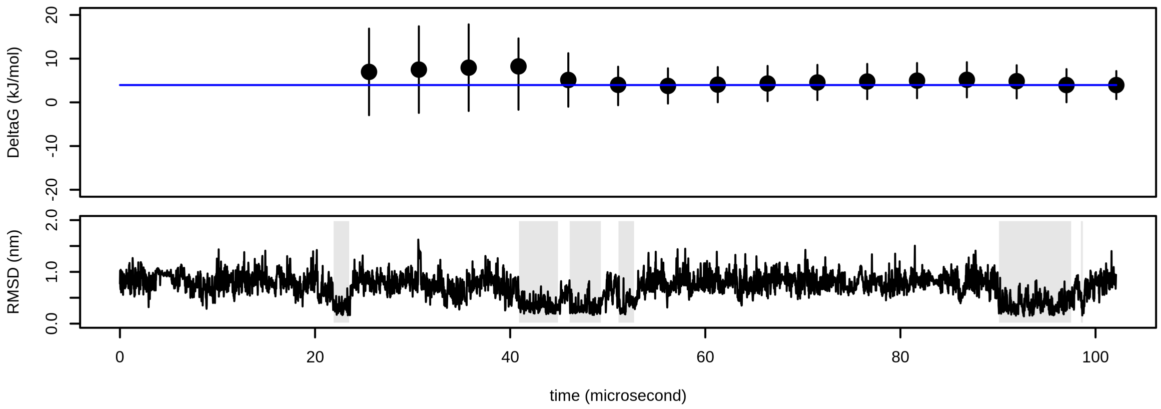

Figure S13: Confidence intervals of folding free energy of BBA (simulation 1). The value of free energy calculated from all simulations is depicted as a blue line.

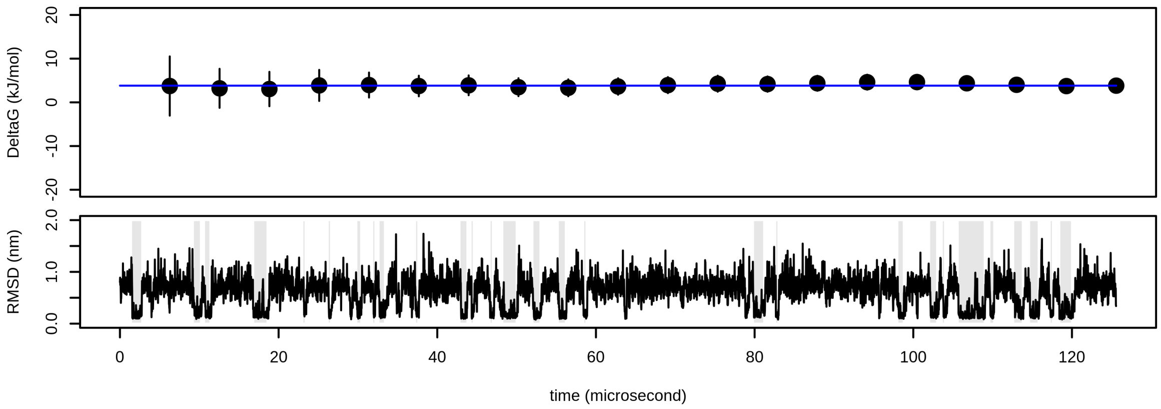

Figure S14: Confidence intervals of folding free energy of villin. The value of free energy calculated for the whole simulation is depicted as a blue line.

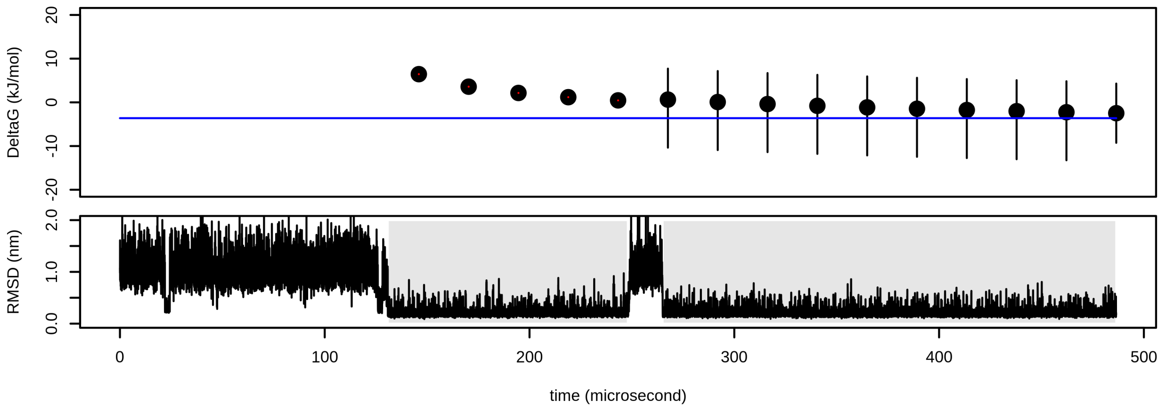

Figure S15: Confidence intervals of folding free energy of WW domain (simulation 0). The value of free energy calculated from all simulations is depicted as a blue line.

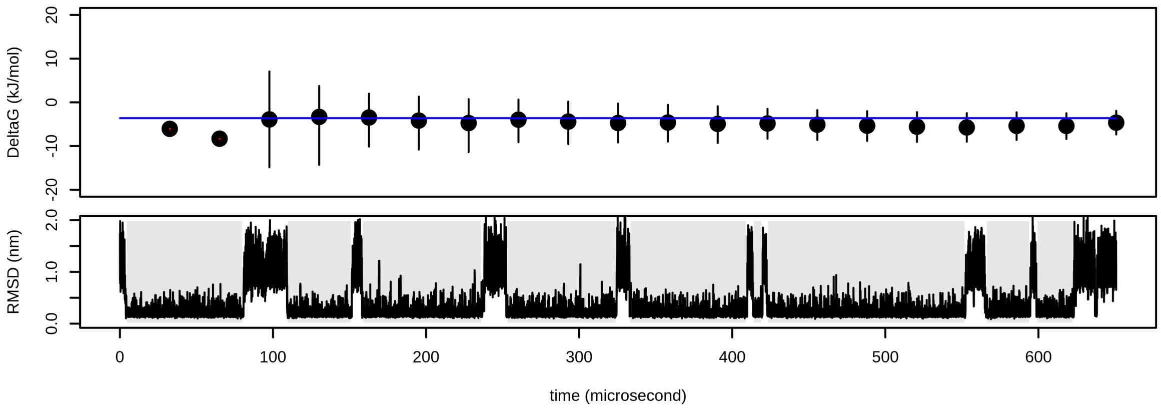

Figure S16: Confidence intervals of folding free energy of WW domain (simulation 1). The value of free energy calculated from all simulations is depicted as a blue line.

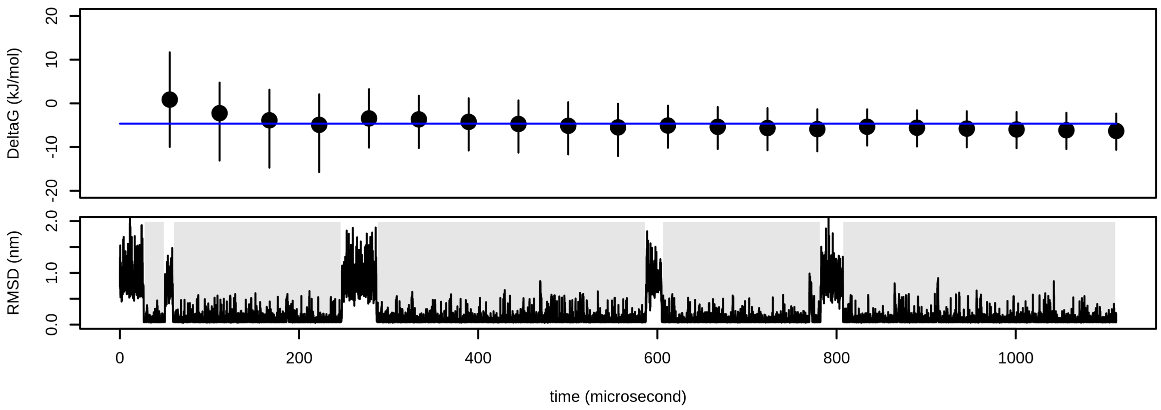

Figure S17: Confidence intervals of folding free energy of NTL9 (simulation 0). The value of free energy calculated from all simulations is depicted as a blue line.

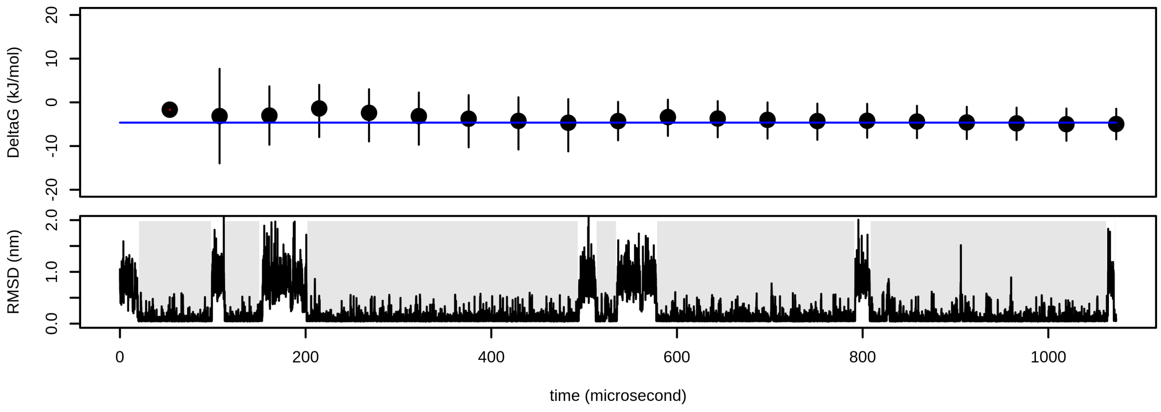

Figure S18: Confidence intervals of folding free energy of NTL9 (simulation 1). The value of free energy calculated from all simulations is depicted as a blue line.

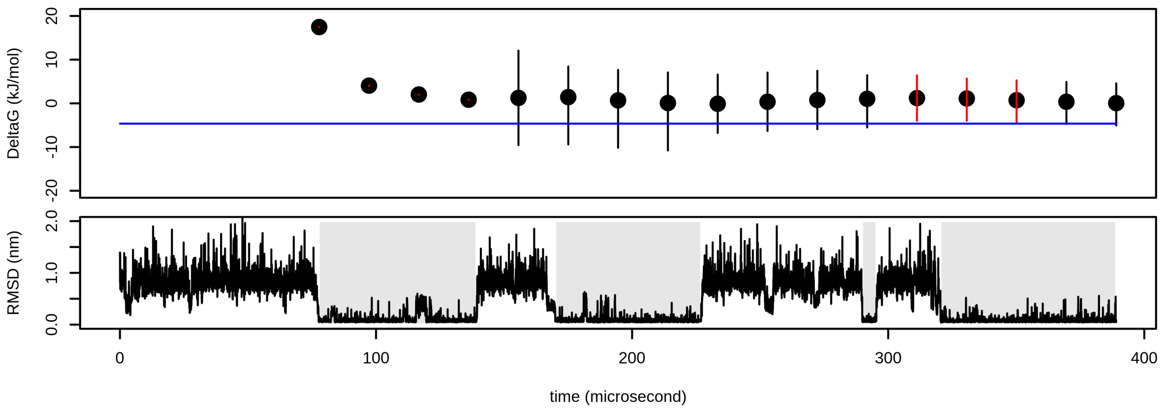

Figure S19: Confidence intervals of folding free energy of NTL9 (simulation 2). The value of free energy calculated from all simulations is depicted as a blue line. Confidence interval that do not span this value are depicted in red.

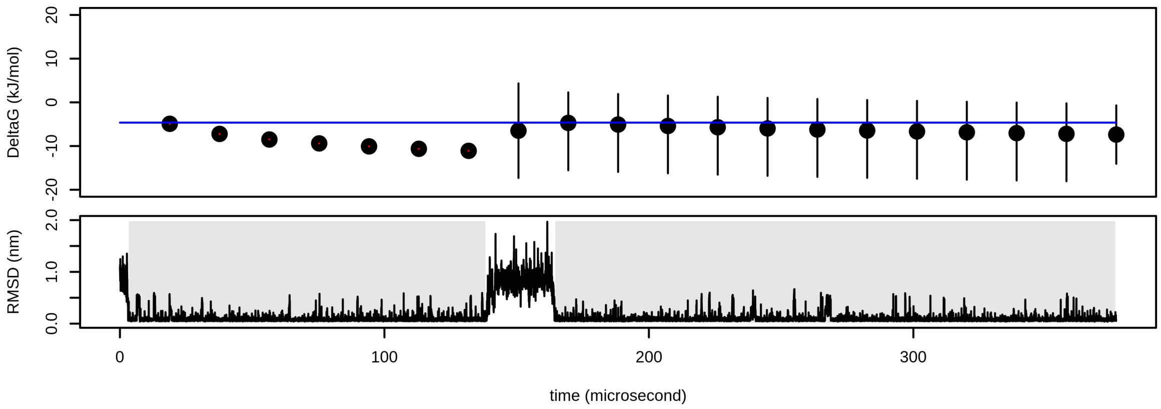

Figure S20: Confidence intervals of folding free energy of NTL9 (simulation 3). The value of free energy calculated from all simulations is depicted as a blue line.

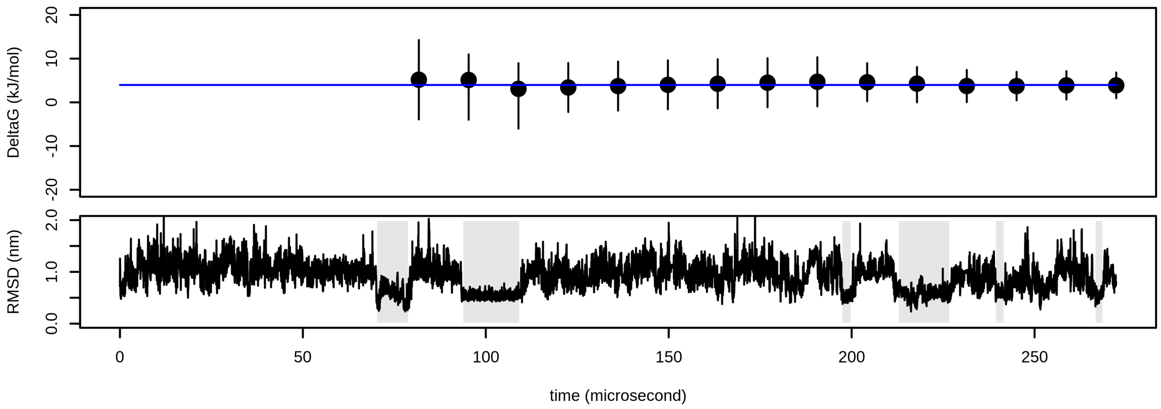

Figure S21: Confidence intervals of folding free energy of BBL (simulation 0). The value of free energy calculated from all simulations is depicted as a blue line.

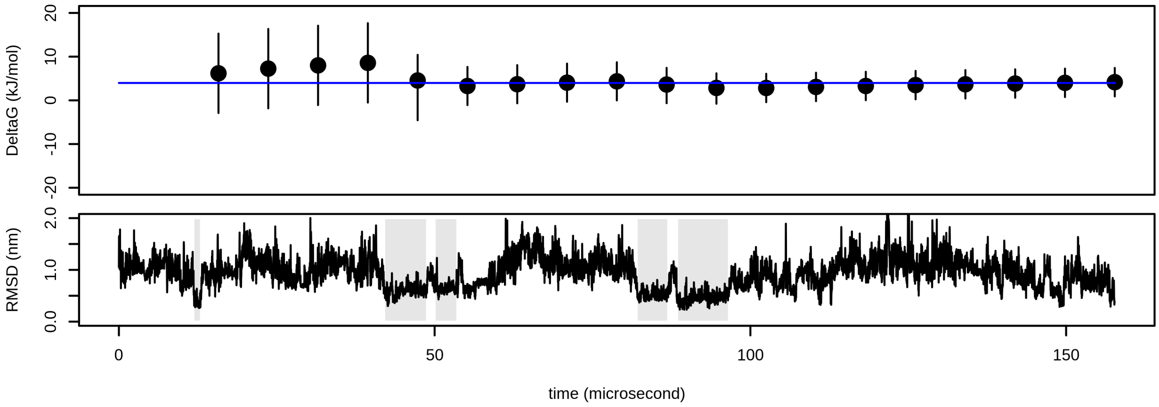

Figure S22: Confidence intervals of folding free energy of BBL (simulation 1). The value of free energy calculated from all simulations is depicted as a blue line.

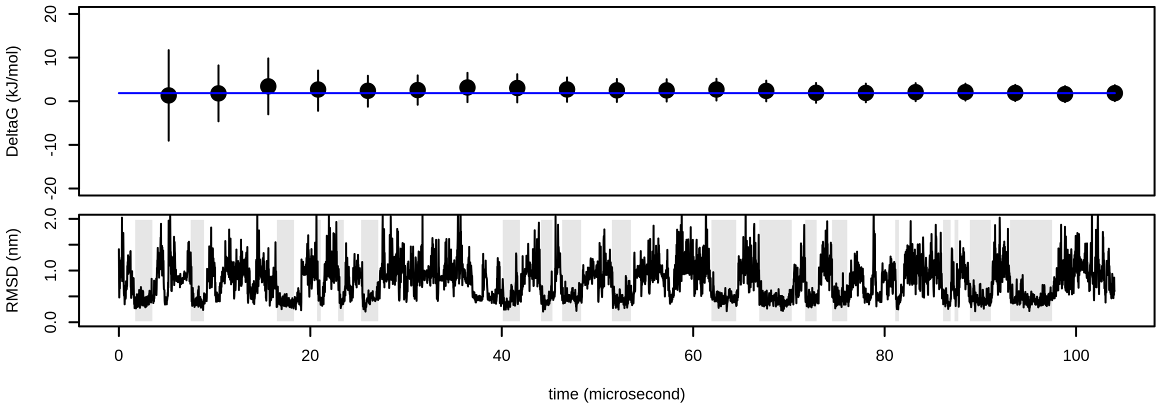

Figure S23: Confidence intervals of folding free energy of Protein B. The value of free energy calculated for the whole simulation is depicted as a blue line.

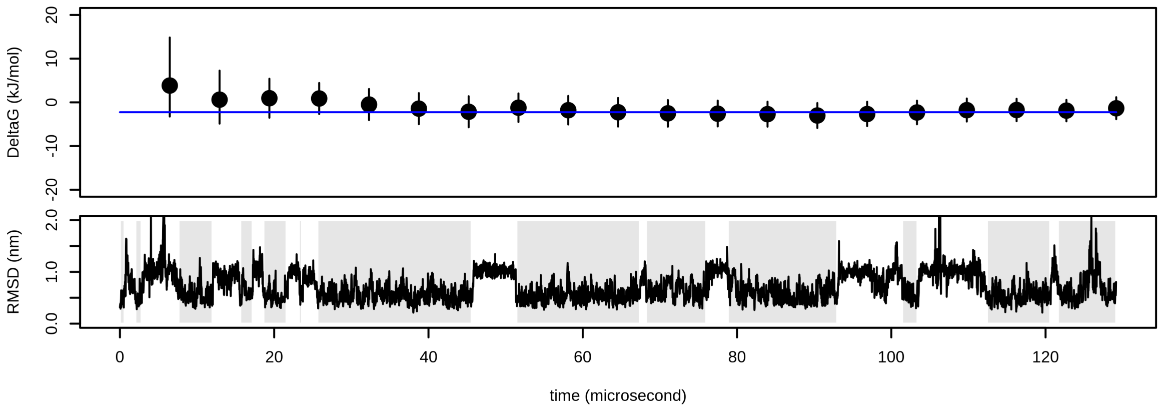

Figure S24: Confidence intervals of folding free energy of Homeodomain (simulation 0). The value of free energy calculated from all simulations is depicted as a blue line.

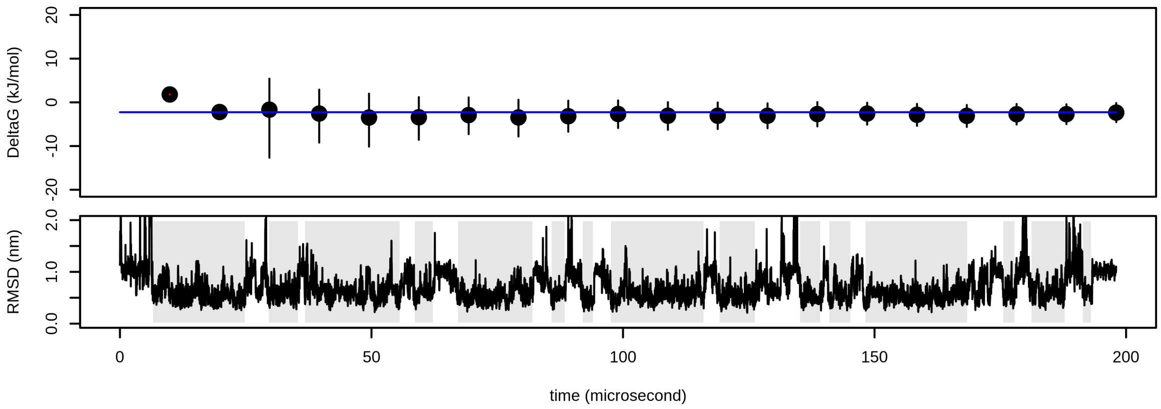

Figure S25: Confidence intervals of folding free energy of Homeodomain (simulation 1). The value of free energy calculated from all simulations is depicted as a blue line.

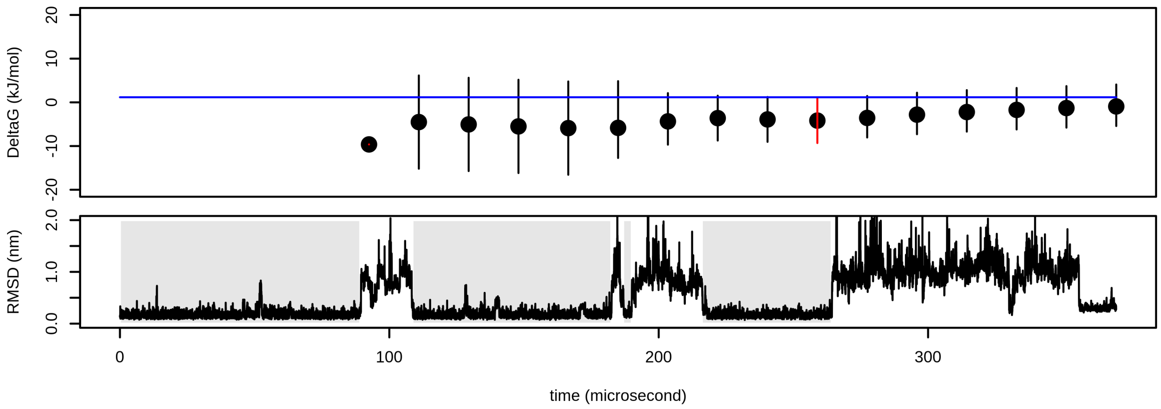

Figure S26: Confidence intervals of folding free energy of Protein G (simulation 0). The value of free energy calculated from all simulations is depicted as a blue line. A confidence interval that does not span this value is depicted in red.

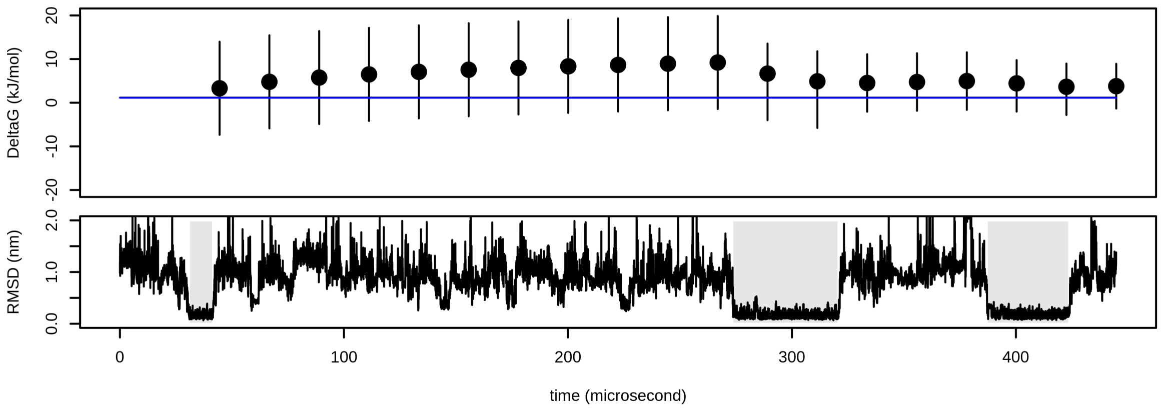

Figure S27: Confidence intervals of folding free energy of Protein G (simulation 1). The value of free energy calculated from all simulations is depicted as a blue line.

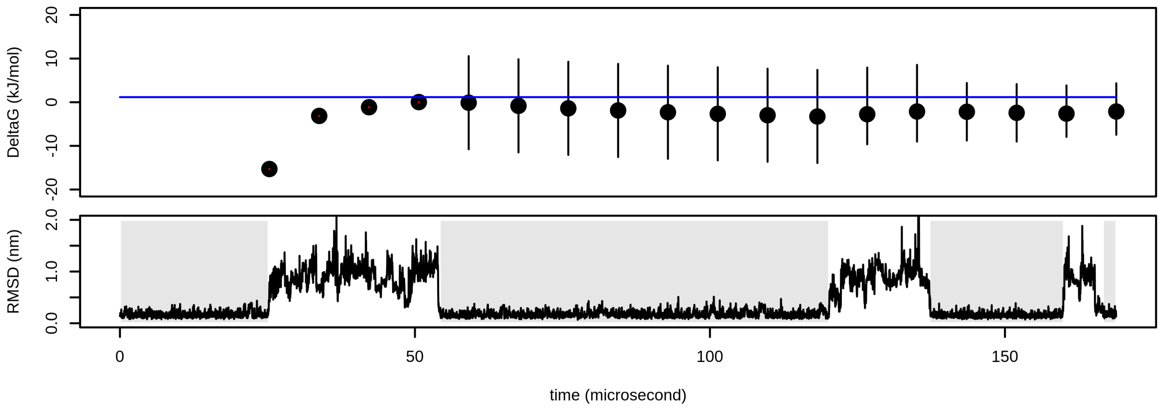

Figure S28: Confidence intervals of folding free energy of Protein G (simulation 2). The value of free energy calculated from all simulations is depicted as a blue line.

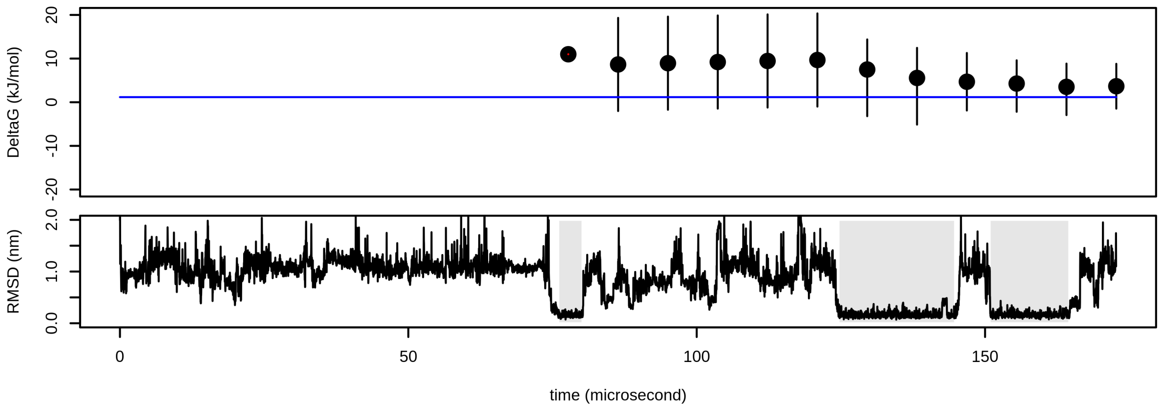

Figure S29: Confidence intervals of folding free energy of Protein G (simulation 3). The value of free energy calculated from all simulations is depicted as a blue line.

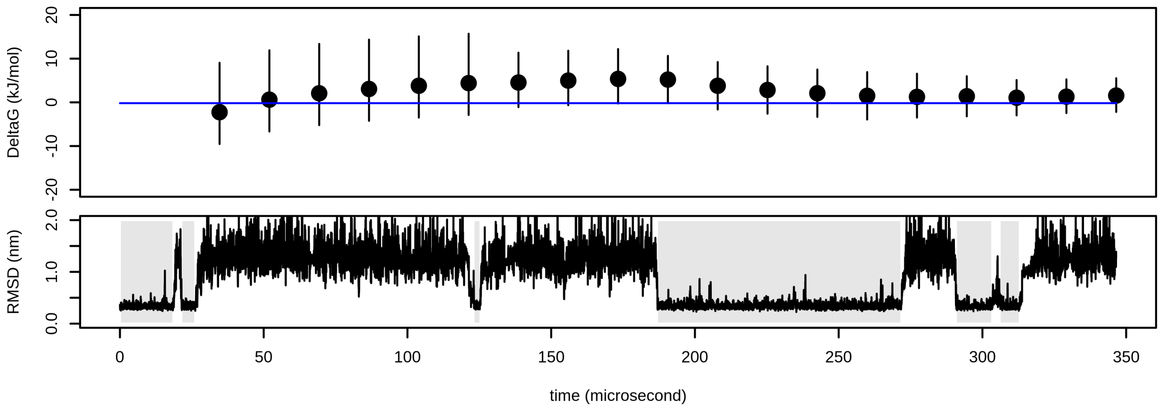

Figure S30: Confidence intervals of folding free energy of α3D (simulation 0). The value of free energy calculated from all simulations is depicted as a blue line.

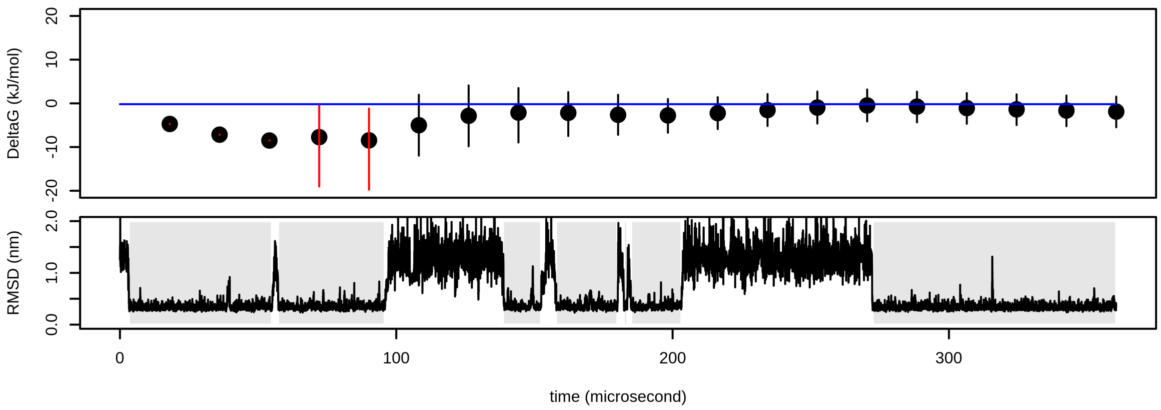

Figure S31: Confidence intervals of folding free energy of α3D (simulation 1). The value of free energy calculated from all simulations is depicted as a blue line. Confidence intervals that do not span this value are depicted in red.

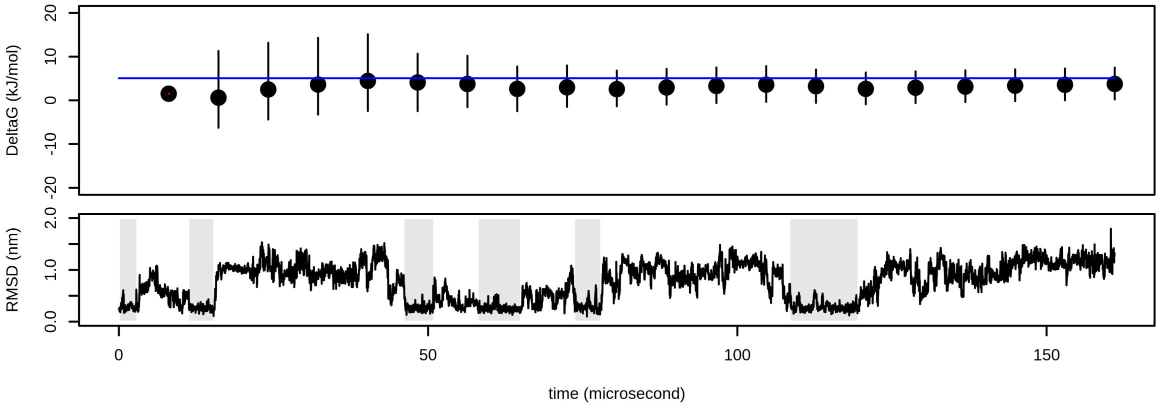

Figure S32: Confidence intervals of folding free energy of λ-repressor (simulation 0). The value of free energy calculated from all simulations is depicted as a blue line.

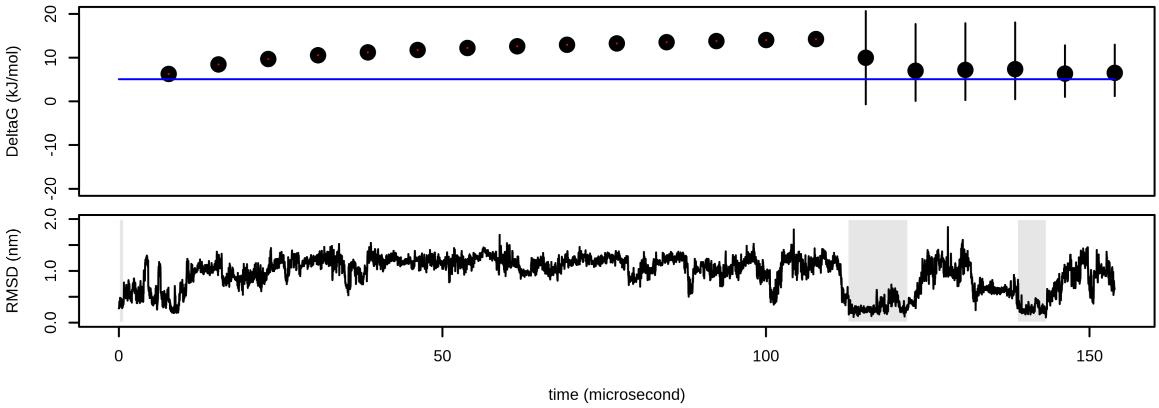

Figure S33: Confidence intervals of folding free energy of λ-repressor (simulation 1). The value of free energy calculated from all simulations is depicted as a blue line.

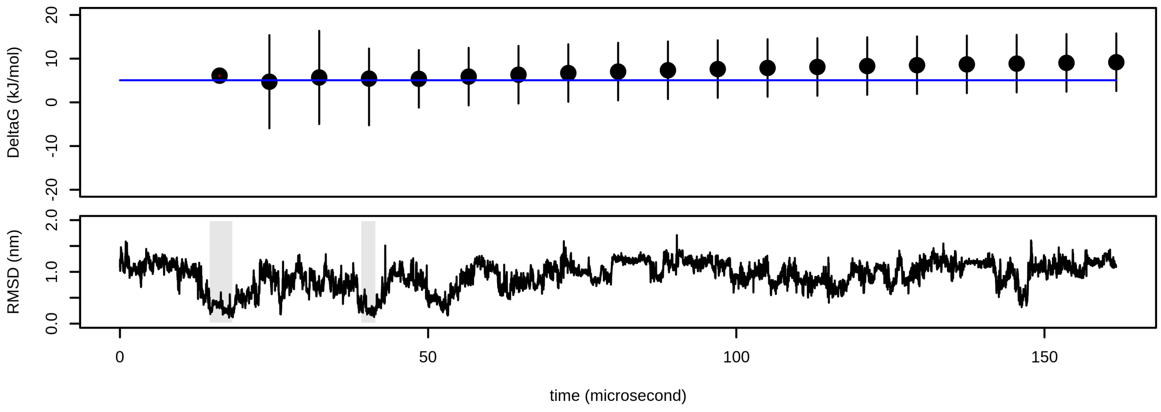

Figure S34: Confidence intervals of folding free energy of λ-repressor (simulation 2). The value of free energy calculated from all simulations is depicted as a blue line.

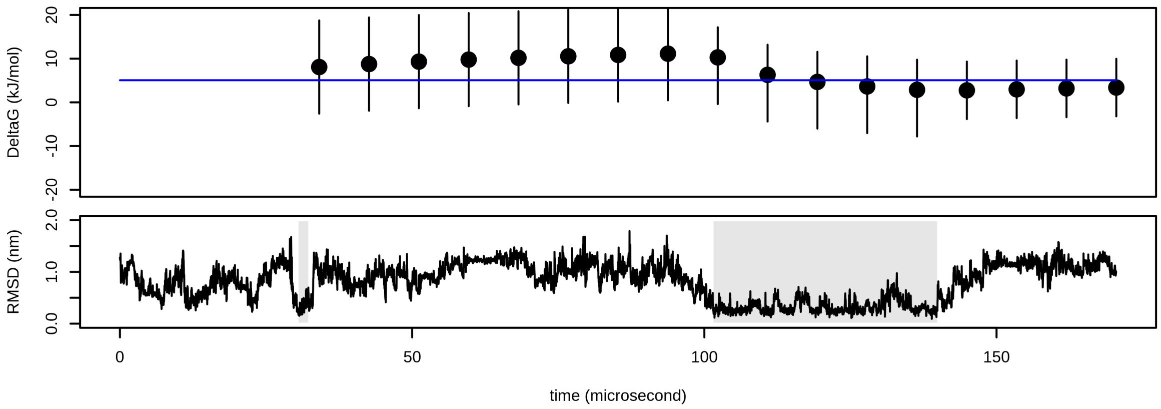

Figure S35: Confidence intervals of folding free energy of λ-repressor (simulation 3). The value of free energy calculated from all simulations is depicted as a blue line.

4 Calculation of rate constant and ΔG_0 from a series of simulations

It is possible to perform n_A simulations starting from the state A until they reach state B and n_B simulations starting from state B until they reach state A. The value of the equilibrium constant can be then estimated as:

$$\hat{K} = \frac{n_A \sum_{i=1}^{n_B} t_{Bi}}{n_B \sum_{j=1}^{n_A} t_{Aj}}, \tag{2}$$

and ΔG_0 can be estimated as $-kT \log \hat{K}$.

However, it is important to either run all simulations until they reach the desired state or to estimate the equilibrium constant as a ratio of the two maximum likelihood estimates of the rate parameters in exponential distributions calculated from the right-censored data :

$$\hat{K} = \frac{n_A(m_B t_{max} + \sum_{j=1}^{n_B} t_j)}{n_B(m_A t_{max} + \sum_{i=1}^{n_A} t_j)}, \tag{3}$$

if some of the simulations do not reach the desired state before time t_{max}.

To illustrate the adaptation of maximum likelihood estimates to right-censored data, let us consider a pair of simulations of the same system starting from the state A. One of the simulations reaches the state B at time t_1. The second reaches the time t_{max} without a transition (i.e. there is an unknown $t_2 > t_{max}$. The probability density function of time t is:

$$f(t,k) = ke^{-kt}, \tag{4}$$

with the cumulative distribution function:

$$F(t,k) = 1 - e^{-kt}. \tag{5}$$

The probability of reaching t_{max} without a transition is:

$$P(t_2 > t_{max}|k) = 1 - (1 - e^{-kt_{max}}) = e^{-kt_{max}}. \tag{6}$$

Maximum likelihood function (the probability of observing the transition in the first system at time t and not observing the transition in the second system before t_{max} is:

$$L(k) = f(t_1, k)P(t_2 > t_{max}|k) = ke^{-kt_1}(1 - F(t_{max}, k)) = ke^{-kt_1}e^{-kt_{max}}. \tag{7}$$

Its logarithm is:

$$\log L(k) = \log k - kt_1 - kt_{max}. \tag{8}$$

Derivative of the logarithm over the rate constant k is set to zero (maximum likelihood) as:

$$\frac{d}{dk}\log L(k) = \frac{1}{k} - t_1 - t_{max} = 0, \tag{9}$$

which leads to:

$$\hat{k} = \frac{1}{t_1 + t_{max}}. \tag{10}$$

For $t_1, ...t_n$ and unknown $t_{n+1}, ..., t_{n+m} > t_{max}$ it can be generalized to:

$$\hat{k} = \frac{n}{\sum_{i=1}^{n} t_i + mt_{max}}. \tag{11}$$

This can be converted to Equation S3 because the equilibrium constant is the product of the rate constants of opposite reactions.

The concept was tested numerically (Figure S36). Two values of t_{max} (1 and 2 in an arbitrary unit) and four numbers of simulations n (5, 10, 20, and 50) were tested. For each of these scenarios, we performed 10,000 simulations. In each simulation, we generated n random values of t with the exponential distribution (rate=1 in an arbitrary unit). Green bars show in how many simulations we observed $t < t_{max}$. For each simulation, we calculated

the value of k (expected to be 1) either using Equation S11 (correct estimator), or by an equation which discards the unfinished simulations:

$$\hat{k} = \frac{n}{\sum_{i=1}^{n} t_i}, \tag{12}$$

(wrong estimator). For each simulation, we calculated the confidence interval of k using the Gamma distribution. Rate of type 1 errors (number of 95% CIs not covering the exact value of $k = 1$) is presented as orange bars. The correct estimator (C) provides a type 1 error rate close to expected 5 %, whereas the wrong estimator (W) provides wrong confidence intervals.

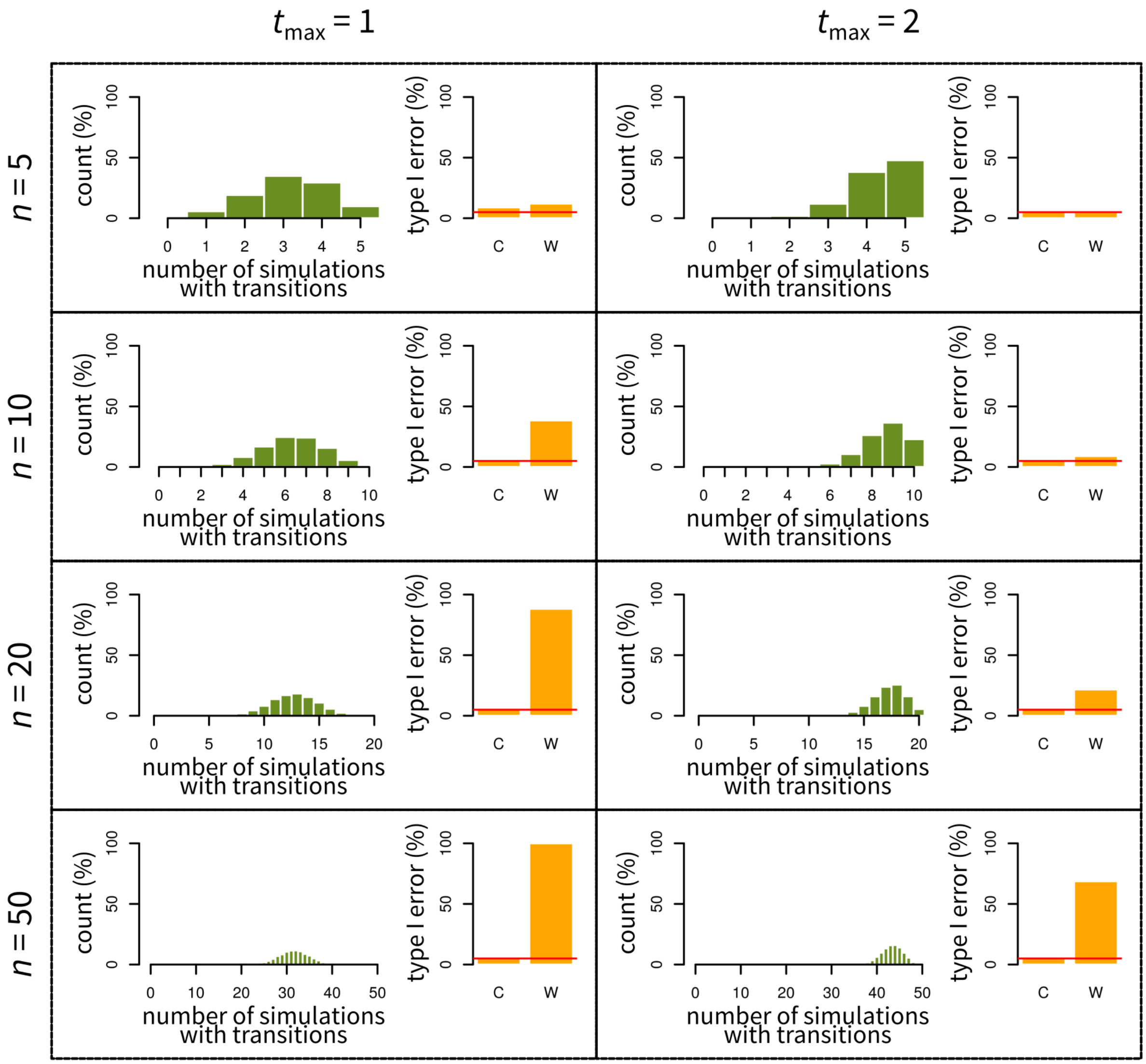

Figure S36: Influence of discarding of unfinished simulations on estimated rate constants (see text for explanation).

5 Calculation of errors in various programming and statistical languages

5.1 R

In R, 95-% CI and standard error for ΔG can be calculated as:

```
nA <- 3       # number of A to B transitions
nB <- 3       # number of B to A transitions
alpha <- 0.05 # value of significance level (0.05 for 95 % CI)
temp <- 300   # temperature in Kelvins
err_top    <-  8.314*temp*log(qf(p=(1-alpha/2), df1=2*nB, df2=2*nA))
err_bottom <- -8.314*temp*log(qf(p=alpha/2, df1=2*nB, df2=2*nA))
se <- 8.314*temp*sqrt(trigamma(nA) + trigamma(nB))
print(err_top/1000)     # height of top errorbar in kJ/mol
print(err_bottom/1000)  # height of bottom errorbar in kJ/mol
print(se/1000)          # standard error in kJ/mol
```

5.2 Python

In Python (with package scipy), 95-% CI and standard error for ΔG can be calculated as:

```
import scipy as sp
from scipy.stats import f
nA = 3          # number of A to B transitions
nB = 3          # number of B to A transitions
alpha = 0.05    # value of significance level (0.05 for 95 % CI)
temp = 300.0    # temperature in Kelvins
err_top    =  8.314*temp*sp.log(f.ppf(q=(1-alpha/2), dfn=2*nB, dfd=2*nA))
err_bottom = -8.314*temp*sp.log(f.ppf(q=alpha/2, dfn=2*nB, dfd=2*nA))
se = 8.314*temp*sp.sqrt(sp.special.polygamma(1, nA) + sp.special.polygamma(1, nB))
print(err_top/1000.0)     # height of top errorbar in kJ/mol
print(err_bottom/1000.0)  # height of bottom errorbar in kJ/mol
print(se/1000.0)          # standard error in kJ/mol
```

5.3 Wolfram Mathematica

In Wolfram Mathematica, 95-% CI and standard error for ΔG can be calculated as:

```
nA = 3;         (* number of A to B transitions *)
nB = 3;         (* number of B to A transitions *)
alpha = 0.05;   (* value of significance level (0.05 for 95 % CI) *)
temp = 300.0;   (* temperature in Kelvins *)
errTop = 8.314*temp*Log[Quantile[FRatioDistribution[2*nB, 2*nA], 1 - alpha/2]];
errBottom = -8.314*temp*Log[Quantile[FRatioDistribution[2*nB, 2*nA], alpha/2]];
se = 8.314*temp*Sqrt[PolyGamma[1, nA] + PolyGamma[1, nB]];
Print[errTop/1000.]     (* height of top errorbar in kJ/mol *)
Print[errBottom/1000.]  (* height of bottom errorbar in kJ/mol *)
Print[se/1000]          (* standard error in kJ/mol *)
```

5.4 Octave

In Octave, 95-% CI and standard error for ΔG can be calculated as:

```
nA = 3          # number of A to B transitions

nB = 3          # number of B to A transitions

alpha = 0.05    # value of significance level (0.05 for 95 % CI)

temp = 300.0    # temperature in Kelvins

errTop = 8.314*temp*log(finv(1 - alpha/2, 2*nB, 2*nA))

errBottom = -8.314*temp*log(finv(alpha/2, 2*nB, 2*nA))

se = 8.314*temp*sqrt(psi(1, nA) + psi(1, nB))

errTop/1000.    # height of top errorbar in kJ/mol

errBottom/1000. # height of bottom errorbar in kJ/mol

se/1000.        # standard error in kJ/mol
```

5.5 Julia

In Julia (with package `Distributions` and `SpecialFunctions`), 95-% CI and standard error for ΔG can be calculated as:

```
using Distributions
using SpecialFunctions
nA = 3         # number of A to B transitions
nB = 3         # number of B to A transitions
alpha = 0.05   # value of significance level (0.05 for 95 % CI)
temp = 300.0   # temperature in Kelvins
d = FDist(2*nB,2*nA)
err_top    =  8.314*temp*log(quantile(d, 1-alpha/2.0))
err_bottom = -8.314*temp*log(quantile(d, alpha/2.0))
se = 8.314*temp*sqrt(trigamma(nA) + trigamma(nB))
print(err_top/1000.0)     # height of top errorbar in kJ/mol
print(err_bottom/1000.0)  # height of bottom errorbar in kJ/mol
print(se/1000.0)          # standard error in kJ/mol
```
